# Impact of concurrent systemic and inhaled corticosteroid use on clinical outcomes in advanced lung cancer patients receiving immune checkpoint inhibitors

**DOI:** 10.1186/s12931-025-03482-5

**Published:** 2026-01-19

**Authors:** Yi Liu, Jiarui Zhang, Linhui Yang, Jiadi Gan, Qi Qi, Wanqin Fang, Huohuo Zhang, Rui Xu, Sha Liu, Jun Yang, Weimin Li, Dan Liu

**Affiliations:** 1 Department of Pulmonary and Critical Care Medicine, West China Hospital, Sichuan University, Chengdu, Sichuan 610041 China; 2 Department of Pulmonary and Critical Care Medicine, Langzhong People’s Hospital, Langzhong, Sichuan 637400 China; 3 State Key Laboratory of Respiratory Health and Multimorbidity, West China Hospital, Chengdu, Sichuan 610041 China; 4 Institute of Respiratory Health, West China Hospital, Sichuan University, Chengdu, Sichuan 610041 China

**Keywords:** Systemic corticosteroids, Inhaled corticosteroids, Immune checkpoint inhibitors, Advanced lung cancer, Efficacy.

## Abstract

**Background:**

Corticosteroids are frequently used during immune checkpoint inhibitor (ICI) treatment, especially in lung cancer patients with comorbidities. Previous studies suggest that systemic corticosteroids (SCS) hinder the effectiveness of ICIs, while the impact of inhaled corticosteroids (ICS) remains unclear. We aimed to examine the association between concurrent SCS and ICS on clinical outcomes in advanced lung cancer patients treated with ICIs.

**Methods:**

This retrospective cohort study enrolled adults with advanced lung cancer who initiated ICIs between September 1 2016 and September 30 2023 at West China Hospital of Sichuan University. Exposure included concurrent SCS, ICS versus no steroid treatment. Clinical outcomes including overall survival (OS), progression-free survival (PFS), and tumor response were assessed. Time-dependent Cox regression models (treating SCS and ICS use as time-varying covariates) were applied to account for immortal-time bias.

**Results:**

Among 368 patients, 122 were SCS users, 51 were ICS users and 195 did not receive corticosteroids. SCS use was associated with inferior PFS (hazard ratio [HR] 1.99; 95% confidence interval [CI], 1.40–2.84; p value < 0.001) and OS (HR 1.77; 95% CI, 1.25–2.51; p value = 0.001), whereas ICS use was not significantly associated with PFS (HR 1.35; 95% CI, 0.66–2.80; p value = 0.412) or OS (HR 1.48; 95% CI, 0.74–2.97; p value = 0.269). Subgroup and sensitivity analyses generally supported the robustness of these findings. In exploratory analyses restricted to SCS users, initiation of SCS within 2 months after ICI start was associated with worse survival.

**Conclusions:**

This study suggests that concurrent SCS use may adversely affect the clinical outcomes of advanced lung cancer patients receiving immunotherapy, whereas ICS use did not appear to compromise ICI efficacy. These findings highlight the critical need for cautious consideration when combining ICIs with systemic corticosteroids and emphasize the *importance* of treating both cancer and lung comorbidity simultaneously.

**Supplementary Information:**

The online version contains supplementary material available at 10.1186/s12931-025-03482-5.

## Introduction

 Immune checkpoint inhibitors (ICIs) have transformed the landscape of oncologic treatment [1]. ICIs, represented by programmed cell death ligand 1 (PD-L1) inhibitors, programmed cell death 1 (PD-1) inhibitors, and cytotoxic T-lymphocyte-associated protein 4 (CTLA-4) inhibitors, have become standard therapies [2, 3]. Emerging data has demonstrated their significant clinical advantages in advanced lung cancer patients [4–7]. However, these benefits were identified exclusively in a subset of patients who showed positive response to ICI therapy [8]. Therefore, there has been a pressing need to identify predictors of ICI response and optimize treatment strategies.

Given the prevalence of lung cancer comorbidities, concerns have been raised regarding concomitant medication use during ICI therapy, which may interact with the immune system [9, 10]. Corticosteroids (CS), including systemic and inhaled corticosteroids, play an indispensable role in clinical practice for comprehensive indications, such as managing immune-related adverse events (irAEs), palliating cancer-related symptoms (anorexia, dyspnea, pain, symptomatic brain metastasis, etc.), and treating comorbid conditions (chronic obstructive pulmonary disease, rheumatic disease, allergies, etc.) [11–13]. Systemic corticosteroids (SCS) have been shown to diminish the ICI effectiveness in advanced lung cancer patients by inducing immunosuppression [11, 14–16]. However, the effect of inhaled corticosteroids (ICS) on clinical outcomes in ICI-treated advanced lung cancer patients remains unclear. Clinical data on ICS in this setting are scarce and mainly derived from retrospective cohorts including heterogeneous advanced cancers. Several studies have reported that baseline inhaled corticosteroid use was not associated with shorter survival in contrast to systemic corticosteroid use, whereas one study observed an association between ICS and an increased risk of checkpoint inhibitor pneumonitis [17–19]. In addition, studies evaluating ICI responses in small-cell lung cancer (SCLC) are limited, as SCLC patients have often been excluded from previous clinical trials. Thus, further studies are needed to elucidate the correlation between CS prescription and clinical outcomes in advanced lung cancer patients.

To address this issue in the literature, this study aimed to examine the association between concomitant corticosteroids (SCS and ICS) and the efficacy of ICIs in advanced lung cancer patients.

## Methods

### Design and study population

This single-center retrospective study was conducted at West China Hospital of Sichuan University, including consecutive patients who initiated ICIs between September 1 2016 and September 30 2023. Eligible participants included adults (age ≥ 18 years) with advanced lung cancer who had been treated with single-agent PD-(L)1 inhibitor (atezolizumab, sintilimab, pembrolizumab, durvalumab, nivolumab, tislelizumab, or camrelizumab) or combined with CTLA-4 inhibitor, ipilimumab. Patients were excluded if they received less than two cycles of PD-(L)1 immunotherapy, or received other concomitant chemotherapy or targeted therapy. Those enrolled in clinical trials potentially receiving placebos were also excluded.

Demographic data and patient characteristics were retrieved from electronic health records. Clinicopathologic characteristics including age, sex, body mass index (BMI), smoking status, comorbidities (recorded based on physician-documented diagnoses in the electronic medical records), ICI use (treatment line, agent used), corticosteroid use (type, indication, route of administration, date of corticosteroid initiation during ICI treatment), histology, sites of metastasis, PD-L1 tumor proportion score (TPS) (evaluated via immunohistochemistry), Eastern Cooperative Oncology Group Performance Status (ECOG PS) at the start of treatment, death, disease progression, and tumor response evaluation, were collected. All participants were followed until death or data lock (April 1 2024), whichever came first. This study received approval from the institutional ethics committee of West China Hospital, Sichuan University (No. 2020 − 232). Due to the retrospective nature of the study, informed consent from participants was waived.

### Exposure and response evaluation

Patients’ pharmacy records were reviewed to determine corticosteroid prescriptions. Concurrent corticosteroid use was defined as any prescription of systemic or inhaled corticosteroids during ICI treatment. Exposures included treatment with systemic corticosteroids or inhaled corticosteroids versus no steroid treatment. The administration of SCS at a dose equivalent to ≥ 10 mg of prednisone during immunotherapy was documented, as doses < 10 mg were generally not included from clinical trials and were considered within the range of physiologic adrenal replacement [20, 21]. Systemic steroids included dexamethasone, methylprednisolone, prednisone, prednisolone, and hydrocortisone. Only SCS administered through oral routes, intravenous, or intramuscular were considered. Systemic corticosteroid indications were classified as cancer-unrelated, cancer-related, and irAEs. Cancer-related indications included cancer-related symptoms (dyspnea, symptomatic brain metastasis, cancer-related pain, spinal cord compression, and superior vena cava syndrome) and premedication for chemotherapy. The main cancer-unrelated indications were chemotherapy or radiation pneumonitis, chronic obstructive pulmonary disease (COPD) exacerbation, pulmonary infection, dermatomyositis and endocrine disorders requiring steroid replacement therapy. irAEs included immune-related pneumonitis, myocarditis, nephritis, thyroiditis, enterocolitis, encephalitis, and dermatologic adverse events. Concurrent ICS use was defined as any prescription of inhaled budesonide, fluticasone, and beclomethasone overlapping with ICI treatment. Intranasal corticosteroids were excluded. The indications for ICS included chronic obstructive pulmonary disease or asthma. The primary outcome was overall survival (OS), defined as the time elapsed between ICI initiation and death of any cause. We also evaluated progression-free survival (PFS), disease control rate (DCR), and objective response rate (ORR). PFS was defined as the time interval from ICI initiation to the earliest occurrence of disease progression or death. DCR was defined as the sum of complete response (CR), partial response (PR), and stable disease (SD), while ORR was defined as CR plus PR. Participants who remained alive and did not experience disease progression were censored at the end of the study (April 1, 2024). Response rates were determined by independent radiologists utilizing the Response Evaluation Criteria in Solid Tumors (RECIST 1.1) [22].

### Statistical analysis

Baseline demographic and characteristics were described as number (percentage) for categorical variables, and median (interquartile range) for continuous variables. Intergroup comparisons were conducted using one-way analysis of variance or the Kruskal-Wallis test for quantitative data, and the Chi-square test or Fisher’s exact test for qualitative variables. Missing data were handled using multiple imputation. To account for potential immortal-time bias, time-dependent Cox regression was applied to estimate hazard ratio (HR) and 95% confidence interval (CI) for PFS and OS, treating corticosteroid exposure (both SCS and ICS) as a time-varying covariate. Variables that were univariately associated with outcomes (*p * < 0.10) were incorporated into the multivariate analysis. Proportional hazards assumptions were assessed using Schoenfeld residuals. Cox regression model was employed as a supplementary contrast analysis. Survival curves were estimated by the Kaplan-Meier method, with group comparisons conducted using the log-rank test. Statistical tests were conducted at a significance threshold of *p* < 0.05 (two-tailed). All analyses were conducted utilizing R software version 4.5.1 and SPSS version 26.0.

## Results

### Patient characteristics

**Fig. 1:**
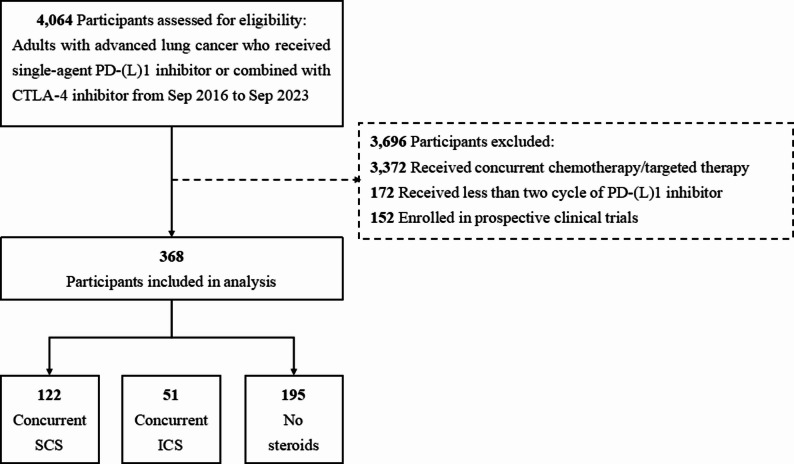
Participants enrollment flow diagram. Abbreviations: PD-1, programmed cell death1; PD-L1, programmed cell death ligand 1; CTLA-4, cytotoxic T-lymphocyte-associated protein 4;SCS, systemic corticosteroids; ICS, inhaled corticosteroids

Among the 4,064 consecutive patients who received PD-(L)1 inhibitor for advanced lung cancer between September 2016 and September 2023, we finally identified 368 participants who met the study criteria. Of 368 patients, 122 received concurrent systemic steroids, 51 received inhaled corticosteroids, and 195 did not receive steroid treatment (Fig. 1). A summary of patients’ demographic and characteristics is presented in Table 1. The median age was 66 years (interquartile range [IQR], 58.0 to 71.1 years), with most patients being male (87.0%), ever smokers (64.9%) and having a baseline ECOG PS of 0–1 (89.7%). Adenocarcinoma (48.4%) was the most frequent histological subtype, followed by squamous cell carcinoma (41.8%), small cell carcinoma (5.2%) and other subtypes (4.6%). ICIs were administered to 46.7% of cases as first line therapy. 81.5% received PD-1 inhibitors and the rest received anti-PD-L1 agents. Bone metastases were present in 23.9% of the participants, brain metastases in 16.8% and liver metastases in 10.9%. Patients’ characteristics, in terms of sex, smoking status, ICI therapy and PD-L1 TPS, were generally well balanced across groups. Notably, concurrent steroid users were older and were more likely to have poor ECOG PS and more respiratory diseases.

**Table 1:** Demographic and characteristic of the study population

Characteristic	Overall (*n* = 368)	No Steroids (*n* = 195)	Concurrent SCS (*n* = 122)	Concurrent ICS (*n* = 51)	*P* value
Age, years	66.0 (58.0, 71.1)	65.0 (57.0, 70.0)	64.3 (57.0, 71.0)	70.2 (64.7, 75.0)	< 0.001
Sex, n (%)					
Male	320 (87.0)	164 (84.1)	107 (87.7)	49 (96.1)	0.074
Female	48 (13.0)	31 (15.9)	15 (12.3)	2 (3.9)	
BMI, kg/m^2^	22.7 (20.5, 25.0)	22.6 (20.5, 25.0)	22.7 (20.3, 24.5)	23.0 (22.0, 25.0)	0.174
Smoking status, n (%)					
Never	129 (35.1)	73 (37.4)	41 (33.6)	15 (29.4)	0.519
Ever	239 (64.9)	122 (62.6)	81 (66.4)	36 (70.6)	
ECOG PS, n (%)					
0–1	330 (89.7)	183 (93.8)	105 (86.1)	42 (82.4)	0.016
≥ 2	38 (10.3)	12 (6.2)	17 (13.9)	19 (17.6)	
Histology, n (%)					
Adenocarcinoma	178 (48.4)	93 (47.7)	66 (54.1)	19 (37.3)	0.007
Squamous	154 (41.8)	87 (44.6)	37 (30.3)	30 (58.8)	
Small cell	19 (5.2)	7 (3.6)	10 (8.2)	2 (3.9)	
Other^a^	17 (4.6)	8 (4.1)	9 (7.4)	0 (0.0)	
PD-L1 TPS, n (%)					
Negative (< 1%)	52 (14.1)	32 (16.4)	18 (14.8)	2 (3.9)	0.072
Positive (≥ 1%)	316 (85.9)	163 (83.6)	104 (85.2)	49 (96.1)	
ICI line, n (%)					
1st line	172 (46.7)	88 (45.1)	59 (48.4)	25(49.0)	0.803
≥ 2nd line	196 (53.3)	107 (54.9)	63 (51.6)	26 (51.0)	
ICI therapy, n (%)					
Anti-PD-1 antibody	300 (81.5)	160 (82.1)	100 (82.0)	40 (78.4)	0.829
Anti-PD-L1 antibody	68 (18.5)	35 (17.9)	22 (18.0)	11 (21.6)	
Brain metastasis, n (%)					
Yes	62 (16.8)	36 (18.5)	24 (19.7)	2 (3.9)	0.028
No	306 (83.2)	159 (81.5)	98 (80.3)	49 (96.1)	
Liver metastasis, n (%)					
Yes	40 (10.9)	23 (11.8)	12 (9.8)	5 (9.8)	0.833
No	328 (89.1)	172 (88.2)	110 (90.2)	46 (90.2)	
Bone metastasis, n (%)					
Yes	88 (23.9)	48 (24.6)	32 (26.2)	8 (15.7)	0.315
No	280 (76.1)	147 (75.4)	90 (73.8)	43 (84.3)	
Comorbidities, n (%)					
Hypertension	97 (26.4)	48 (24.6)	29 (23.8)	20 (39.2)	0.079
COPD	83 (22.6)	18 (9.2)	20 (16.4)	45 (88.2)	< 0.001
Diabetes	57 (15.5)	30 (15.4)	21 (17.2)	6 (11.8)	0.664
Cardiovascular diseases	43 (11.7)	24 (12.3)	11 (9.0)	8 (15.7)	0.426
CKD	11 (3.0)	6 (3.1)	3 (2.5)	2 (3.9)	0.871
NLR	3.4 (2.3, 5.0)	3.2 (2.1, 5.1)	3.5 (2.3, 5.4)	3.6 (2.5, 4.8)	0.252
PLR	162.7 (113.3, 257.9)	155.7 (107.8, 243.9)	185.7 (117.5, 308.6)	155.0 (115.9, 211.7)	0.083
LDH, IU/L	191.0 (162.0, 211.0)	190.0 (157.0, 207.0)	197.9 (166.0, 225.0)	179.0 (157.0, 200.0)	0.073
Corticosteroid indication, n (%)					
Cancer-related^b^	-	-	79 (64.8)	-	
Cancer-unrelated^c^	-	-	16 (13.1)	-	
irAEs	-	-	27 (22.1)	-	
COPD	-	-	-	49 (96.1)	
Asthma	-	-	-	2 (3.9)	

Data are given as n (%) or median (IQR). Significant at *p* < 0.05

*Abbreviations*: *IQR* Interquartile range, *SCS* Systemic corticosteroids, *ICS* Inhaled corticosteroids, *BMI* Body mass index, *ECOG PS* Eastern Cooperative Oncology Group Performance Status, *PD-L1* Programmed cell death ligand 1, *TPS* Tumor proportion score,* ICI* Immune checkpoint inhibitor, *PD-1* Programmed cell death 1, *COPD* Chronic obstructive pulmonary disease, *CKD* Chronic kidney disease, *NLR* Neutrophil-to-lymphocyte ratio, *PLR* Platelet-to-lymphocyte ratio, *LDH* Lactate dehydrogenase, *irAEs* Immune-related adverse events

^a^Other histologic types include sarcomatoid carcinoma, pleomorphic carcinoma, lymphoepithelioma-like carcinoma and large cell neuroendocrine carcinoma

^b^Cancer-related indications include cancer-related symptoms and premedication for chemotherapy. Cancer-related symptoms include dyspnea, symptomatic brain metastasis, cancer-related pain, spinal cord compression and superior vena cava syndrome

^c^The main cancer-unrelated indications were chemotherapy or radiation pneumonitis, COPD exacerbation, pulmonary infection, dermatomyositis and endocrine disorders requiring steroid replacement therapy

Regarding corticosteroid indications, 79 patients (64.8%) received concurrent SCS for cancer-related indications, 16 (13.1%) for cancer-unrelated indications, and 27 (22.1%) for irAEs. 49 patients (96.1%) received concurrent ICS for COPD and 2 (3.9%) for asthma. The timing and detailed indication for SCS administration are depicted in Table S1. Overall, 47 patients (38.5%) initiated SCS within 2 months after ICI initiation, whereas 75 (61.5%) started SCS ≥ 2 months after ICI initiation. The most common cancer-related indications were cancer-related symptoms (42.7%), and the main cancer-unrelated indications were chemotherapy or radiation pneumonitis (5.0%). Immune-related pneumonitis represented the most frequent irAE-related indication (11.5%). Dexamethasone was the most frequently used steroid (54.9%), followed by methylprednisolone and prednisone.

### Clinical outcomes

**Fig. 2:**
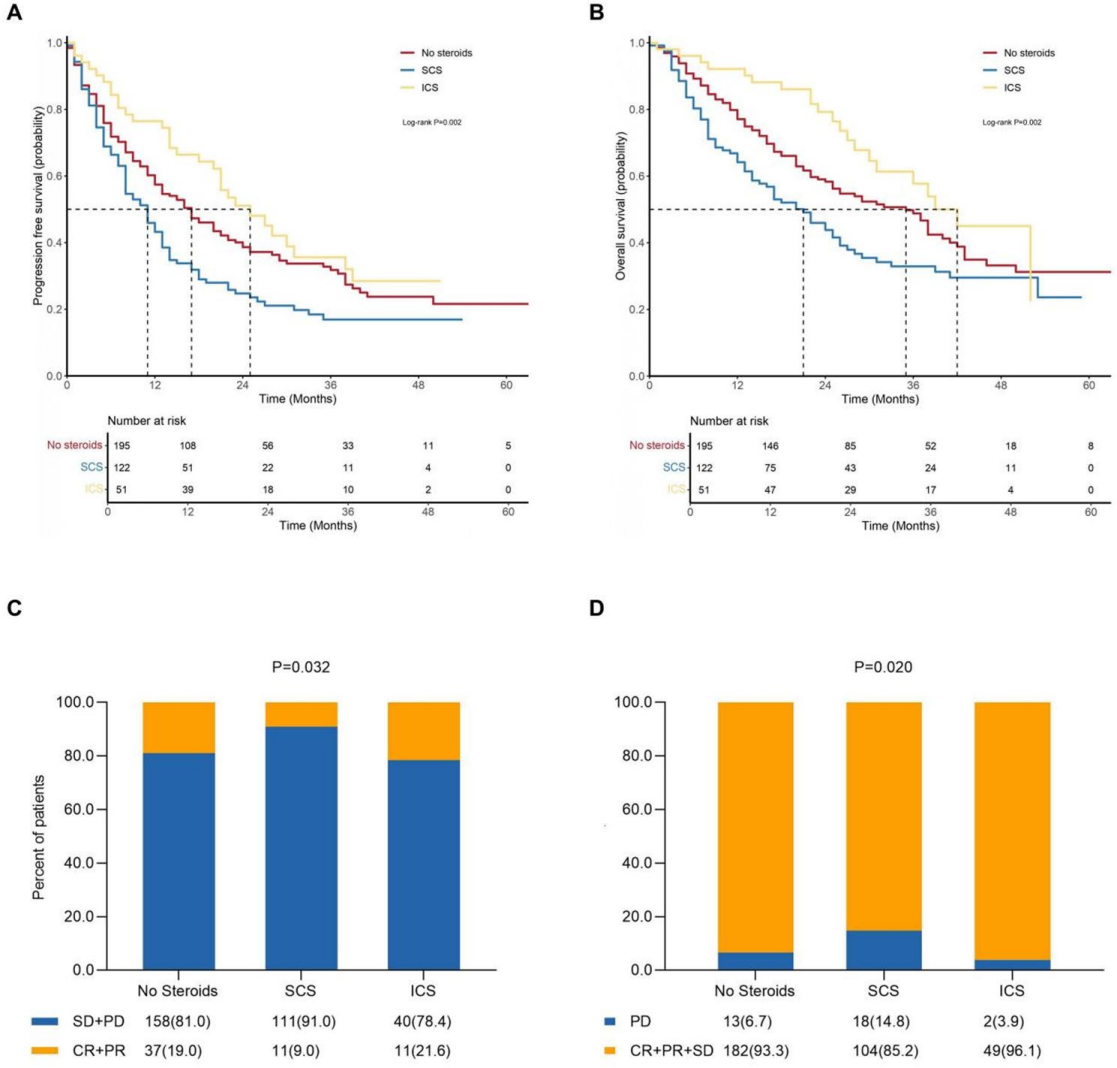
Clinical outcomes of patients treated with concurrent SCS, ICS, or no steroids. **A** Kaplan-Meier curves for progression-free survival. **B** Kaplan-Meier curves for overall survival. **C** Objective response rate. **D** Disease control rate. Abbreviations: SCS, systemic corticosteroids; ICS, inhaled corticosteroids; HR, hazard ratio; CI, confidence interval; CR, complete response; PR, partial response; SD, stable disease; PD, progressive disease

The median follow-up duration was 21.0 months in the entire cohort. Kaplan-Meier analysis showed significant differences in PFS across the three groups (log-rank p value = 0.002; Fig. 2A). The median PFS was 11.0 months in the SCS group, 17.0 months in the no steroids group, and 25.0 months in the ICS group. For overall survival, the median OS was 21.0 months in the SCS group, 35.0 months in the no steroids group, and 42.0 months in the ICS group (log-rank p value = 0.002; Fig. 2B). The ORR was 16.0%, while DCR was 91.0% in the total population. Significant differences regarding ORR (no steroids vs SCS vs ICS: 19.0% vs. 9.0% vs. 21.6%; p value = 0.032; Fig. 2C) and DCR (no steroids vs SCS vs ICS: 93.3% vs. 85.2% vs. 96.1%; p value = 0.020; Fig. 2D) were also detected between groups.

Time-dependent Cox regression results for PFS and OS are presented in Table 2. After adjusting for age, BMI, smoking status, ICI agent, PD-L1 TPS, comorbidities, laboratory parameters, and history of liver and bone metastasis, multivariate analysis indicated that SCS use was associated with inferior PFS (HR 1.99; 95% CI, 1.40–2.84; p value < 0.001) and OS (HR 1.77; 95% CI, 1.25–2.51; p value = 0.001). Whereas, ICS use did not show a statistically significant association with PFS (HR 1.35; 95% CI, 0.66–2.80; p value = 0.412) or OS (HR 1.48; 95% CI, 0.74–2.97; p value = 0.269). Cardiovascular diseases, bone metastasis and liver metastasis were significantly associated with an increased risk of disease progression or death. In contrast, PD-L1 positivity was found to be correlated with significantly improved PFS and OS. Multivariate Cox regression analysis yielded consistent results, which indicated that SCS use was linked to worse survival outcomes. ICS use remained not significantly associated with PFS but was associated with improved OS (HR 0.57; 95% CI, 0.35–0.93; p value = 0.024) (Table S2). This discrepancy may reflect residual immortal-time bias. Nevertheless, the general consistency between models reinforces the stability of the findings. Additional sensitivity analysis indicated that ICI agent did not change the associations between SCS or ICS exposure after multivariable adjustment (Table S3).

**Table 2:** Univariate and multivariate time-dependent Cox regression analyses for PFS and OS of the study population

Factor	Category	PFS				OS			
		Univariate analysis		Multivariate analysis		Univariate analysis		Multivariate analysis	
		HR (95% CI)	*P*	HR (95% CI)	*P*	HR (95% CI)	*P*	HR (95% CI)	*P*
Age	≥ 65 y vs. <65 y	1.02 (0.80–1.31)	0.865			1.32 (0.99–1.75)	0.057	1.20 (0.88–1.64)	0.252
Sex	Male vs. female	0.78 (0.55–1.10)	0.161			1.12 (0.74–1.70)	0.584		
BMI	≥ 24 vs. <24	0.75 (0.58–0.98)	0.037	0.84 (0.63–1.10)	0.206	0.53 (0.39–0.73)	< 0.001	0.56 (0.40–0.78)	0.001
Smoking status	Ever vs. never	0.96 (0.74–1.25)	0.775			1.29 (0.96–1.74)	0.092	1.36 (1.00-1.87)	0.051
ECOG PS	≥ 2 vs. 0–1	1.01 (0.68–1.51)	0.944			1.02 (0.65–1.60)	0.943		
Squamous	Yes vs. no	0.93 (0.72–1.19)	0.559			1.07 (0.81–1.43)	0.617		
PD-L1 positive^*^	Yes vs. no	0.60 (0.43–0.83)	0.002	0.58 (0.42–0.80)	0.001	0.69 (0.47–0.99)	0.043	0.57 (0.39–0.84)	0.004
ICI line	1st vs. ≥ 2nd	0.95 (0.74–1.22)	0.700			1.03 (0.78–1.36)	0.860		
ICI therapy	PD-1 vs. PD-L1	1.30 (0.91–1.86)	0.152			1.84 (1.16–2.93)	0.010	1.42 (0.88–2.29)	0.152
Brain metastasis	Yes vs. no	1.08 (0.78–1.49)	0.662			1.20 (0.84–1.71)	0.305		
Liver metastasis	Yes vs. no	2.26 (1.59–3.22)	< 0.001	1.68 (1.11–2.55)	0.014	1.86 (1.25–2.76)	0.002	1.58 (1.03–2.42)	0.038
Bone metastasis	Yes vs. no	1.80 (1.37–2.37)	< 0.001	1.54 (1.14–2.08)	0.005	1.86 (1.38–2.52)	< 0.001	1.62 (1.17–2.25)	0.004
Hypertension	Yes vs. no	0.98 (0.74–1.29)	0.869			1.07 (0.78–1.47)	0.661		
COPD	Yes vs. no	0.94 (0.70–1.26)	0.677			1.13 (0.81–1.56)	0.473		
Diabetes	Yes vs. no	1.05 (0.75–1.49)	0.770			1.21 (0.83–1.77)	0.323		
Cardiovascular disease	Yes vs. no	1.48 (1.03–2.13)	0.035	1.71 (1.18–2.48)	0.004	1.94 (1.32–2.85)	0.001	2.21 (1.47–3.32)	< 0.001
CKD	Yes vs. no	1.15 (0.57–2.33)	0.690			0.95 (0.42–2.15)	0.910		
NLR	Ratio	1.03 (1.00-1.07)	0.057	1.02 (0.97–1.07)	0.410	1.03 (1.00-1.07)	0.070	1.03 (0.99–1.07)	0.119
PLR	Ratio	1.00 (1.00–1.00)	0.016	1.00 (1.00–1.00)	0.459	1.00 (1.00–1.00)	0.103		
LDH	UI/L	1.00 (1.00–1.00)	0.006	1.00 (1.00–1.00)	0.565	1.00 (1.00–1.00)	0.018	1.00 (1.00–1.00)	0.929
SCS (time-varying)	SCS vs. no steroids	2.01 (1.42–2.84)	< 0.001	1.99 (1.40–2.84)	< 0.001	1.89 (1.36–2.62)	< 0.001	1.77 (1.25–2.51)	0.001
ICS (time-varying)	ICS vs. no steroids	1.17 (0.58–2.40)	0.657	1.35 (0.66–2.80)	0.412	1.46 (0.74–2.86)	0.272	1.48 (0.74–2.97)	0.269

*Abbreviations*: *PFS* Progression-free survival, *OS* Overall survival, *HR* Hazard ratio, *CI* Confidence interval,* BMI* Body mass index, *ECOG PS* Eastern Cooperative Oncology Group Performance Status, *PD-L1* Programmed cell death ligand 1, *ICI* Immune checkpoint inhibitor, *PD-1* Programmed cell death 1, *COPD* Chronic obstructive pulmonary disease, *CKD* Chronic kidney disease, *NLR* Neutrophil-to-lymphocyte ratio, *PLR* Platelet-to-lymphocyte ratio, *LDH* Lactate dehydrogenase, *SCS* Systemic corticosteroids, *ICS* Inhaled corticosteroids

^*^PD-L1 positive was defined as PD-L1 TPS ≥ 1%

### Subgroup analysis

**Fig. 3:**
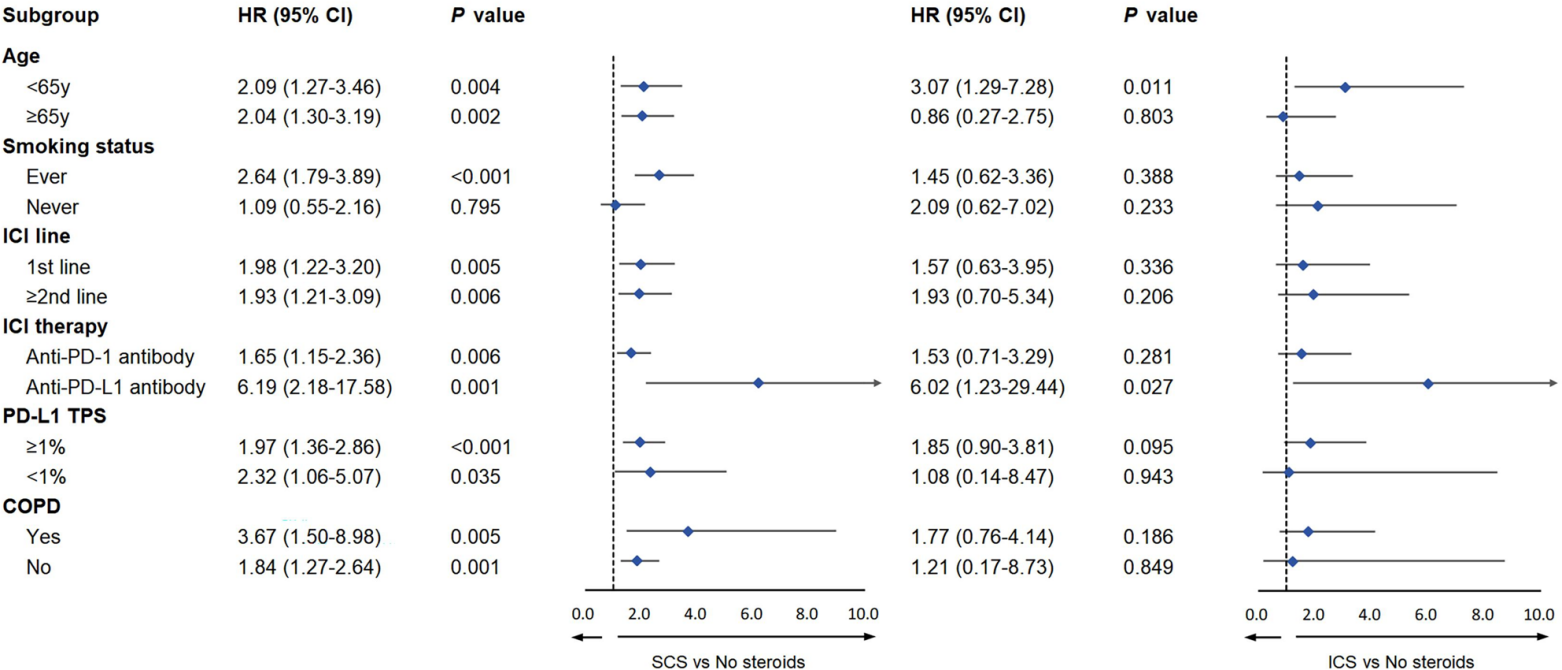
Forest plots of subgroup analyses for OS according to corticosteroid exposure based on time-dependent Cox regression. Subgroup analyses were based on time-dependent Cox models adjusted for age and PD-L1 status. Abbreviations: HR, hazard ratio; CI, confidence interval; ICI, immune checkpoint inhibitor; PD-1, programmed cell death 1; PD-L1, programmed cell death ligand 1; TPS, tumor proportion score; COPD, chronic obstructive pulmonary disease; SCS, systemic corticosteroids; ICS, inhaled corticosteroids

We further examined the association in populations with different characteristics, using multivariable time-dependent Cox models with SCS and ICS included as time-varying covariates (Fig. 3). Subgroup analyses for overall survival demonstrated the robustness of clinical outcomes in general. The detrimental association between SCS exposure and OS was generally observed across all subgroups. In contrast, ICS exposure did not show a clear or consistent association with OS, reflecting the limited number of ICS users within each subgroup. In an exploratory analysis restricted to patients receiving concurrent SCS, we examined the impact of SCS timing, type and indication on ICI efficacy. Compared with SCS initiation ≥ 2 months, initiation < 2 months was linked to decreased overall survival (median OS 11.0 vs. 24.0 months; p value = 0.024; Figure S1A), whereas PFS did not differ significantly between the two groups (median PFS 8.0 vs. 12.0 months; p value = 0.669; Figure S1D). SCS indications showed no significant difference in OS (median OS 22.0 vs. 21.0 months vs. 13.0 months; p value = 0.930; Figure S1B) and PFS (median PFS 11.0 vs. 11.0 months vs. 9.0 months; p value = 0.954; Figure S1E). Additionally, OS (median OS 17.0 vs. 22.0 months; p value = 0.824; Figure S1C) and PFS (median PFS 11.0 vs. 10.0 months; p value = 0.495; Figure S1F) were comparable between patients receiving short- or intermediate-acting versus long-acting SCS. Patients receiving dexamethasone had poor survival compared to the steroids-naïve population (HR 1.43; 95% CI, 1.01–2.04; p value = 0.044; Figure S2). The sensitivity analyses restricted to COPD patients (Table S4) and non-small cell lung cancer patients (Table S5) yielded results consistent with those of the overall cohort, confirming the robustness of our findings.

## Discussion

Immune checkpoint inhibitors are currently important anti-cancer approaches. Although ICIs have demonstrated remarkable effectiveness in lung cancer treatment, not all patients exhibit favorable responses [23, 24]. Therefore, the identification of predictors of ICI response is desperately needed. In this retrospective study, we conducted time-dependent Cox analysis to address the potential immortal-time bias. After adjusting for clinically relevant variables, SCS exposure was consistently associated with inferior PFS and OS, as well as a lower tumor response rate, indicating that SCS may attenuate the therapeutic benefit of ICIs. In contrast, ICS prescription did not adversely affect survival outcomes, even in patients with COPD who frequently require ICS therapy. These associations were robust to challenge in subgroup analyses. In exploratory analyses restricted to SCS users, early SCS initiation (< 2 months after ICI start) was associated with shorter OS, whereas SCS indication and SCS type were not significantly related to PFS or OS.

Given the prevalence of lung cancer comorbidities, there are growing public concerns over the use of concomitant medication during ICI treatment which may interfere with the immune system [9, 25, 26]. Systemic corticosteroids are widely used and play an essential role in oncologic treatment for various indications. It has raised the concern that SCS may theoretically undermine the efficacy of immunotherapy due to their immunosuppressive properties, such as impairing T-lymphocyte proliferation and differenciation [27, 28]. Several studies have consistently shown the adverse effect of systemic steroids on survival in ICI-treated lung cancer patients [14, 29–31]. A recent meta-analysis indicated that corticosteroids negatively affect both mortality and progression in patients with non-small cell lung cancer (NSCLC) receiving ICIs [11]. After adjusting for potential confounders, our time-dependent Cox analysis of advanced lung cancer patients indicated that SCS exposure was associated with worse survival outcomes, which was in accordance with these findings. The supplementary Cox regression further confirmed the negative effect of SCS, supporting the robustness of this association despite different model specifications. To further address potential heterogeneity caused by inclusion of SCLC cases, we performed a sensitivity analysis restricted to NSCLC patients, and the results were consistent with those of the overall cohort, supporting the robustness of our conclusions. Consequently, the use of systemic corticosteroids should be considered cautiously during immunotherapy.

However, not all SCS use is detrimental. Previous studies have reported that SCS administration for irAEs and cancer-unrelated symptoms did not correlate with lower OS in NSCLC patients undergoing immunotherapy [11, 14, 15]. To further refine the impact of SCS, we explored the timing, types and indications of SCS prescription. In line with prior retrospective studies [32], we found that patients who initiated SCS < 2 months after ICI start had shorter OS, whereas PFS did not differ significantly between early and later SCS initiation. By contrast, SCS indication and SCS type were not significantly associated with OS or PFS in our cohort. These findings should be interpreted with caution, as early SCS use likely reflects more aggressive disease, higher symptom burden and poorer baseline prognosis, and confounding by indication cannot be excluded. Our research provides insights into the negative effect of concurrent medication use on clinical outcomes in patients receiving immunotherapy. On the basis of these findings, it may be prudent to delay SCS initiation and explore alternative pharmacologic strategies to treat cancer-related indications in patients receiving ICI treatment.

As essential medicines listed by the World Health Organization, inhaled corticosteroids are among the most prescribed medications worldwide. Although previous studies have determined that systemic corticosteroids negatively affected the prognosis of lung cancer patients receiving ICIs, evidence regarding the association between ICS and ICI efficacy remains limited. In contrast to existing literature, we found that inhaled corticosteroid administration did not appear to influence survival or tumor response rate in advanced lung cancer patients treated with ICIs, suggesting that ICS may be a viable alternative for patients undergoing ICIs in certain cases. While the traditional Cox model indicated a survival benefit associated with ICS, this association disappeared after applying a time-dependent Cox model. The attenuation of statistical significance suggests that the apparent OS benefit observed in the conventional Cox analysis may be partly attributable to residual immortal-time bias. One possible explanation for this discrepancy is that ICS was predominantly prescribed to patients with COPD, a population that has consistently shown improved responses to ICI therapy. Prior studies have shown that COPD could alter the immune cell composition and enhance the efficacy of ICIs by modulating the tumor microenvironment [33–35]. Additionally, inhaled corticosteroids are known to exert potent anti-inflammatory effects in the airways by binding to the glucocorticoid receptor and repressing NF-κB and activator protein-1-driven transcription of multiple inflammatory genes, leading to reduced production of pro-inflammatory cytokines and chemokines such as IL-8 and TNF-α and attenuation of neutrophilic airway inflammation. In patients with COPD, ICS therapy has been shown to decrease airway inflammatory burden, including reductions in sputum neutrophils and neutrophil-associated mediators, and to improve markers of airway inflammation [36]. By dampening chronic airway inflammation and epithelial injury in the bronchial tree while producing relatively low systemic glucocorticoid exposure, ICS could theoretically help maintain a more favorable pulmonary immune milieu during PD-(L)1 blockade. In our cohort, ICS use might not have a detrimental effect on PFS and OS, which is in line with previous retrospective studies [17, 18]. Prospective studies with detailed characterization of airway disease, ICS exposure and immune-related toxicity are warranted. Furthermore, ICS can reduce the frequency of exacerbations in comorbid conditions such as asthma and COPD, maintaining better lung function and enhancing patient tolerance to treatment. In our study, standardized ICS protocols were administered to patients with COPD in accordance with clinical guidelines. To further address COPD-related confounding, we conducted a sensitivity analysis restricted to COPD patients. The time-dependent Cox analysis demonstrated that ICS use did not compromise the efficacy of ICIs in the COPD subgroup. Therefore, our findings add to the evidence base that ICS does not adversely affect ICI efficacy, and emphasize the necessity of simultaneously managing both cancer and lung among ICI-treated individuals with advanced lung cancer.

To our knowledge, this study is the first to evaluate the impact of both systemic and inhaled corticosteroids on the efficacy of ICIs among patients with advanced lung cancer in China. Our findings provide valuable insights into concomitant corticosteroid use during ICI treatment, which can inform the decision-making in clinical practice.

Several limitations of this study need to be acknowledged. Firstly, this retrospective single-center study may be subject to selection bias and unmeasured confounding that may not be fully mitigated despite multivariable adjustment. Therefore, further study with a larger sample size and a prospective design is needed to validate these findings. Secondly, our study concentrated on patients who received PD-(L)1 inhibitors monotherapy, and those treated with fewer than two cycles or in combination with chemotherapy or targeted therapy were excluded. This limits the generalizability of our findings to broader populations. Thirdly, detailed information regarding the cumulative dose, tapering patterns, and duration of systemic corticosteroid therapy could not be reliably quantified because these data were documented inconsistently in real-world clinical practice, with frequent intermittent or overlapping prescriptions. Therefore, corticosteroid exposure was analyzed based on the timing of initiation relative to ICI treatment, which was the most consistently recorded and clinically interpretable measure available. Fourthly, our cohort included both NSCLC and SCLC, which differ in clinical behavior and underlying biology, but the limited number of SCLC cases precluded robust histology-specific analyses. Thus, our findings should be interpreted primarily in the context of advanced NSCLC and extrapolated to SCLC with caution. In addition, irAEs were not systematically captured, and data on the exact timing, organ involvement, and grade were incomplete. Consequently, we were unable to robustly evaluate their impact on ICI efficacy. Moreover, information on tumor mutational burden and lung function test data were not consistently available. Therefore, COPD severity could not be uniformly classified, and residual confounding related to COPD severity cannot be excluded. Ultimately, although standardized prescription criteria and a sensitivity analysis adjusting for ICI agent yielded results consistent with the main analyses, heterogeneity of immunotherapy strategies and residual confounding related to ICI type cannot be entirely excluded.

## Conclusions

In summary, this retrospective study demonstrated that concurrent systemic corticosteroid use was significantly associated with inferior PFS, OS, and tumor response in advanced lung cancer patients treated with ICIs. By contrast, the prescription of inhaled corticosteroids showed no detrimental impact on ICI efficacy, even in patients with COPD who frequently require ICS therapy. These findings underscore the importance of careful consideration when administering systemic corticosteroids during ICI treatment and emphasize the necessity for treating both cancer and lung simultaneously. Further studies involving larger sample sizes are warranted to validate and expand these findings to other populations.

## Supplementary Information

Supplementary Material 1.

Supplementary Material 2.

## Data Availability

Research data can be accessed upon justified request by contacting the corresponding author.

## Abbreviations

BMI: Body mass index
CTLA-4: Cytotoxic T-lymphocyte-associated protein 4
CS: Corticosteroids
COPD: Chronic obstructive pulmonary disease
CKD: Chronic kidney disease
CR: Complete response
CI: Confidence interval
DCR: Disease control rate
ECOG PS: Eastern Cooperative Oncology Group Performance Status
HR: Hazard ratio
ICIs: Immune checkpoint inhibitor
ICS: Inhaled corticosteroids
IQR: Interquartile range
irAEs: Immune-related adverse events
LDH: Lactate dehydrogenase
NSCLC: Non-small cell lung cancer
NLR: Neutrophil-to-lymphocyte ratio
OS: Overall survival
ORR: Objective response rate
PFS: Progression-free survival
PLR: Platelet-to-lymphocyte ratio
PR: Partial response
PD: Progressive disease
PD-1: Programmed cell death 1
PD-L1: Programmed cell death ligand 1
RECIST: Response Evaluation Criteria in Solid Tumors
SCS: Systemic corticosteroids
SD: Stable disease
SCLC: Small-cell lung cancer
TPS: Tumor proportion score
