# Supplementary material for: Impact of concurrent systemic and inhaled corticosteroid use on clinical outcomes in advanced lung cancer patients receiving immune checkpoint inhibitors
Source: Respir Res. 2026 Jan 19;27:59. doi: 10.1186/s12931-025-03482-5 (PMC12896340; doi:10.1186/s12931-025-03482-5)
Supplement: Supplementary file 1 — Supplementary Material 1. [file 12931_2025_3482_MOESM1_ESM.docx]

**Table S1 Characteristic of concurrent systemic steroids treatment.**

| **Characteristic** | **No. of patients (%)** |
| --- | --- |
| Time from ICI initiation to steroids treatment |  |
| <2 months | 47 (38.5) |
| ≥2 months | 75 (61.5) |
| Steroids indication |  |
| Cancer-related^a^ | 79 (64.8) |
| Cancer-related symptoms | 52 (42.7) |
| Premedications of chemotherapy | 27 (22.1) |
| Cancer-unrelated | 16 (13.1) |
| Chemotherapy or radiation pneumonitis | 6 (5.0) |
| Pulmonary infection | 5 (4.1) |
| Endocrine disorders^b^ | 2 (1.6) |
| Dermatomyositis | 2 (1.6) |
| COPD exacerbation | 1 (0.8) |
| irAEs | 27 (22.1) |
| Immune-related pneumonitis | 14 (11.5) |
| Immune-related myocarditis | 5 (4.1) |
| Immune-related enterocolitis | 3 (2.5) |
| Immune-related thyroiditis | 2 (1.6) |
| Immune-related dermatologic adverse events | 1 (0.8) |
| Immune-related encephalitis | 1 (0.8) |
| Immune-related nephritis | 1 (0.8) |
| Steroid type |  |
| Dexamethasone | 67 (54.9) |
| Methylprednisolone | 32 (26.2) |
| Prednisone | 23 (18.9) |
| Data are given as n (%).  Abbreviations: ICI, immune checkpoint inhibitor; COPD, chronic obstructive pulmonary disease; irAE, immune-related adverse event.  ^a^Cancer-related indications include cancer-related symptoms and premedication for chemotherapy. Cancer-related symptoms include dyspnea, symptomatic brain metastasis, cancer-related pain, spinal cord compression and superior vena cava syndrome.  ^b^Endocrine disorders include adrenal insufficiency and thyroid dysfunction requiring steroid replacement therapy. | |

**Table S2 Univariate and multivariate Cox regression analyses for PFS and OS of the study population.**

| **Factor** | **Category** | **PFS** | | | | **OS** | | | |
| --- | --- | --- | --- | --- | --- | --- | --- | --- | --- |
|  |  | **Univariate analysis** | | **Multivariate analysis** | | **Univariate analysis** | | **Multivariate analysis** | |
|  |  | HR (95% CI) | *P* | HR (95% CI) | *P* | HR (95% CI) | *P* | HR (95% CI) | *P* |
| Age | ≥65 y vs. <65 y | 1.02 (0.80-1.31) | 0.860 |  |  | 1.31 (0.99-1.74) | 0.060 | 1.26 (0.92-1.73) | 0.143 |
| Sex | Male vs. female | 0.78 (0.55-1.11) | 0.172 |  |  | 1.12 (0.74-1.70) | 0.585 |  |  |
| BMI | ≥24 vs. <24 | 0.76 (0.58-0.99) | 0.040 | 0.79 (0.60-1.04) | 0.086 | 0.54 (0.39-0.74) | <0.001 | 0.53 (0.38-0.75) | <0.001 |
| Smoking status | Ever vs. never | 0.96 (0.75-1.25) | 0.780 |  |  | 1.29 (0.96-1.74) | 0.096 | 1.38 (1.00-1.88) | 0.047 |
| ECOG PS | ≥2 vs. 0-1 | 1.02 (0.68-1.52) | 0.929 |  |  | 1.02 (0.65-1.60) | 0.941 |  |  |
| Squamous | Yes vs. no | 0.93 (0.72-1.19) | 0.560 |  |  | 1.07 (0.81-1.42) | 0.633 |  |  |
| PD-L1 positive^*^ | Yes vs. no | 0.61 (0.44-0.84) | 0.003 | 0.62 (0.45-0.87) | 0.005 | 0.69 (0.48-1.00) | 0.047 | 0.60 (0.41-0.88) | 0.008 |
| ICI line | 1st vs ≥2nd | 0.96 (0.75-1.23) | 0.720 |  |  | 1.03 (0.78-1.36) | 0.854 |  |  |
| ICI therapy | PD-1 vs. PD-L1 | 1.30 (0.91-1.85) | 0.157 |  |  | 1.83 (1.15-2.91) | 0.011 | 1.32 (0.82-2.13) | 0.257 |
| Brain metastasis | Yes vs. no | 1.07 (0.78-1.49) | 0.672 |  |  | 1.20 (0.84-1.71) | 0.309 |  |  |
| Liver metastasis | Yes vs. no | 2.20 (1.54-3.14) | <0.001 | 1.77 (1.17-2.68) | 0.007 | 1.84 (1.24-2.73) | 0.003 | 1.74 (1.12-2.70) | 0.014 |
| Bone metastasis | Yes vs. no | 1.78 (1.35-2.34) | <0.001 | 1.54 (1.14-2.08) | 0.005 | 1.85 (1.37-2.50) | <0.001 | 1.70 (1.23-2.35) | 0.001 |
| Hypertension | Yes vs. no | 0.98 (0.74-1.30) | 0.882 |  |  | 1.07 (0.79-1.47) | 0.659 |  |  |
| COPD | Yes vs. no | 0.94 (0.70-1.26) | 0.692 |  |  | 1.13 (0.81-1.56) | 0.474 |  |  |
| Diabetes | Yes vs. no | 1.05 (0.75-1.49) | 0.775 |  |  | 1.21 (0.83-1.77) | 0.328 |  |  |
| Cardiovascular disease | Yes vs. no | 1.47 (1.02-2.12) | 0.037 | 1.69 (1.17-2.45) | 0.005 | 1.92 (1.31-2.82) | 0.001 | 2.14 (1.42-3.22) | <0.001 |
| CKD | Yes vs. no | 1.15 (0.57-2.33) | 0.693 |  |  | 0.95 (0.42-2.15) | 0.907 |  |  |
| NLR | Ratio | 1.03 (1.00-1.07) | 0.066 | 1.03 (0.98-1.08) | 0.281 | 1.03 (1.00-1.07) | 0.074 | 1.03 (1.00-1.07) | 0.082 |
| PLR | Ratio | 1.00 (1.00-1.00) | 0.021 | 1.00 (1.00-1.00) | 0.802 | 1.00 (1.00-1.00) | 0.111 |  |  |
| LDH | UI/L | 1.00 (1.00-1.00) | 0.008 | 1.00 (1.00-1.00) | 0.925 | 1.00 (1.00-1.01) | 0.020 | 1.00 (1.00-1.00) | 0.684 |
| SCS use | SCS vs. no steroids | 1.42 (1.09-1.85) | 0.011 | 1.40 (1.07-1.85) | 0.016 | 1.43 (1.06-1.93) | 0.018 | 1.41 (1.03-1.93) | 0.031 |
| ICS use | ICS vs. no steroids | 0.75 (0.51-1.11) | 0.147 | 0.75 (0.50-1.12) | 0.155 | 0.66 (0.41-1.06) | 0.084 | 0.57 (0.35-0.93) | 0.024 |
| Abbreviations: PFS, progression-free survival; OS, overall survival; HR, hazard ratio; CI, confidence interval; BMI, body mass index; ECOG PS, Eastern Cooperative Oncology Group Performance Status; PD-L1, programmed cell death ligand 1; ICI, immune checkpoint inhibitor; PD-1, programmed cell death 1; COPD, chronic obstructive pulmonary disease; CKD, chronic kidney disease; NLR, neutrophil-to-lymphocyte ratio; PLR, platelet-to-lymphocyte ratio; LDH, lactate dehydrogenase; SCS, systemic corticosteroids; ICS, inhaled corticosteroids.  ^*^PD-L1 positive was defined as PD-L1 TPS ≥1%. | | | | | | | | | |

**Table S3 Multivariate time-dependent Cox regression analysis for PFS and OS including ICI agent.**

| **Factor** | **Category** | **PFS** | | **OS** | |
| --- | --- | --- | --- | --- | --- |
|  |  | HR (95% CI) | *P* | HR (95% CI) | *P* |
| Age | ≥65 y vs. <65 y | 1.07 (0.82-1.40) | 0.614 | 1.21 (0.89-1.66) | 0.230 |
| BMI | ≥24 vs. <24 | 0.85 (0.64-1.13) | 0.275 | 0.57 (0.41-0.80) | 0.001 |
| Smoking status | Ever vs. never | 1.09 (0.82-1.43) | 0.559 | 1.38 (1.01-1.89) | 0.046 |
| ICI therapy | PD-1 vs. PD-L1 | 1.12 (0.77-1.64) | 0.554 | 1.45 (0.89-2.34) | 0.135 |
| PD-L1 positive^*^ | Yes vs. no | 0.56 (0.40-0.78) | 0.001 | 0.57 (0.39-0.83) | 0.003 |
| Liver metastasis | Yes vs. no | 1.69 (1.12-2.57) | 0.013 | 1.58 (1.02-2.42) | 0.038 |
| Bone metastasis | Yes vs. no | 1.53 (1.13-2.07) | 0.007 | 1.60 (1.15-2.23) | 0.005 |
| Cardiovascular disease | Yes vs. no | 1.70 (1.16-2.49) | 0.006 | 2.23 (1.48-3.36) | <0.001 |
| NLR | Ratio | 1.02 (0.97-1.07) | 0.535 | 1.02 (0.97-1.07) | 0.479 |
| PLR | Ratio | 1.00 (1.00-1.00) | 0.331 | 1.00 (1.00-1.00) | 0.573 |
| LDH | UI/L | 1.00 (1.00-1.00) | 0.534 | 1.00 (1.00-1.00) | 0.949 |
| SCS (time-varying) | SCS vs. no steroids | 1.99 (1.39-2.84) | <0.001 | 1.77 (1.25-2.50) | 0.001 |
| ICS (time-varying) | ICS vs. no steroids | 1.37 (0.66-2.82) | 0.399 | 1.49 (0.74-2.98) | 0.265 |
| Abbreviations: PFS, progression-free survival; OS, overall survival; HR, hazard ratio; CI, confidence interval; BMI, body mass index; ICI, immune checkpoint inhibitor; PD-1, programmed cell death 1; PD-L1, programmed cell death ligand 1; NLR, neutrophil-to-lymphocyte ratio; PLR, platelet-to-lymphocyte ratio; LDH, lactate dehydrogenase; SCS, systemic corticosteroids; ICS, inhaled corticosteroids.  *PD-L1 positive was defined as PD-L1 TPS ≥1%. | | | | | |

**Table S4 Multivariate time-dependent Cox regression analysis for PFS and OS in the COPD group.**

| **Factor** | **Category** | **PFS** | | **OS** | |
| --- | --- | --- | --- | --- | --- |
|  |  | HR (95% CI) | *P* | HR (95% CI) | *P* |
| Age | ≥65 y vs. <65 y | 1.05 (0.57-1.94) | 0.866 | 0.90 (0.47-1.74) | 0.753 |
| PD-L1 positive^*^ | Yes vs. no | 0.69 (0.33-1.45) | 0.325 | 0.67 (0.30-1.50) | 0.333 |
| SCS (time-varying) | SCS vs. no steroids | 1.63 (0.53-5.00) | 0.390 | 3.67 (1.50-8.98) | 0.005 |
| ICS (time-varying) | ICS vs. no steroids | 1.16 (0.50-2.71) | 0.731 | 1.77 (0.76-4.14) | 0.186 |
| Abbreviations: PFS, progression-free survival; OS, overall survival; COPD, chronic obstructive pulmonary disease; HR, hazard ratio; CI, confidence interval; PD-L1, programmed cell death ligand 1; SCS, systemic corticosteroids; ICS, inhaled corticosteroids.  *PD-L1 positive was defined as PD-L1 TPS ≥1%. | | | | | |

**Table S5 Multivariate time-dependent Cox regression analysis for PFS and OS in the NSCLC group.**

| **Factor** | **Category** | **PFS** | | **OS** | |
| --- | --- | --- | --- | --- | --- |
|  |  | HR (95% CI) | *P* | HR (95% CI) | *P* |
| Age | ≥65 y vs. <65 y | 1.00 (0.77-1.31) | 0.984 | 1.28 (0.93-1.75) | 0.128 |
| BMI | ≥24 vs. <24 | 0.82 (0.62-1.10) | 0.181 | 0.53 (0.37-0.74) | <0.001 |
| PD-L1 positive^*^ | Yes vs. no | 0.59 (0.42-0.83) | 0.002 | 0.63 (0.43-0.93) | 0.020 |
| Liver metastasis | Yes vs. no | 1.79 (1.19-2.69) | 0.006 | 1.65 (1.07-2.54) | 0.023 |
| Bone metastasis | Yes vs. no | 1.52 (1.12-2.06) | 0.008 | 1.66 (1.19-2.32) | 0.003 |
| Cardiovascular disease | Yes vs. no | 1.71 (1.17-2.49) | 0.006 | 2.20 (1.46-3.31) | <0.001 |
| NLR | Ratio | 1.01 (0.96-1.06) | 0.709 | 1.03 (0.98-1.08) | 0.306 |
| PLR | Ratio | 1.00 (1.00-1.00) | 0.196 | 1.00 (1.00-1.00) | 0.654 |
| LDH | UI/L | 1.00 (1.00-1.00) | 0.354 | 1.00 (1.00-1.00) | 0.861 |
| SCS (time-varying) | SCS vs. no steroids | 1.98 (1.38-2.84) | <0.001 | 1.72 (1.20-2.46) | 0.003 |
| ICS (time-varying) | ICS vs. no steroids | 1.31 (0.64-2.71) | 0.462 | 1.42 (0.71-2.84) | 0.324 |
| Abbreviations: PFS, progression-free survival; OS, overall survival; HR, hazard ratio; CI, confidence interval; NSCLC, non-small cell lung cancer; BMI, body mass index; PD-L1, programmed cell death ligand 1; NLR, neutrophil-to-lymphocyte ratio; PLR, platelet-to-lymphocyte ratio; LDH, lactate dehydrogenase; SCS, systemic corticosteroids; ICS, inhaled corticosteroids.  *PD-L1 positive was defined as PD-L1 TPS ≥1%. | | | | | |

**Figure S1 Kaplan-Meier curves of OS and PFS in the concurrent SCS group by SCS timing, indication and type.**

Abbreviations: PFS, progression-free survival; OS, overall survival; irAE, immune-related adverse event.

**Figure S2 Systemic steroid type and overall survival.**


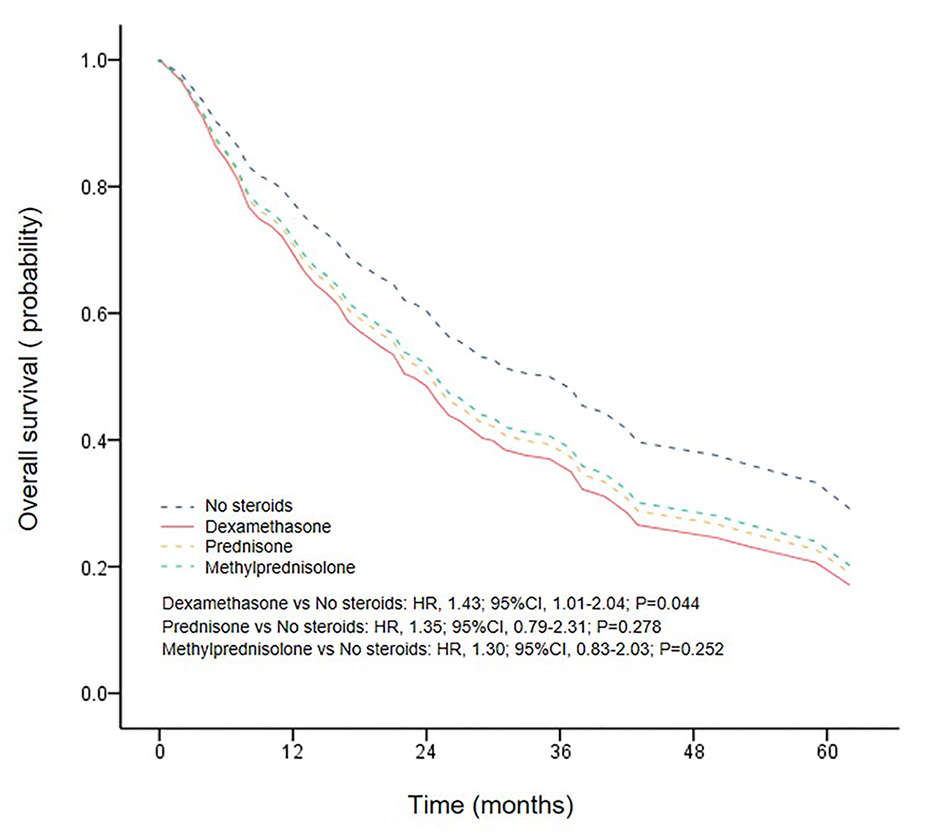


Abbreviations: HR, hazard ratio; CI, confidence interval.
