# Supplementary material for: Impact of concurrent systemic and inhaled corticosteroid use on clinical outcomes in advanced lung cancer patients receiving immune checkpoint inhibitors
Source: Respir Res. 2026 Jan 19;27:59. doi: 10.1186/s12931-025-03482-5 (PMC12896340; doi:10.1186/s12931-025-03482-5)
Supplement: Supplementary file 2 — Supplementary Material 2. [file 12931_2025_3482_MOESM2_ESM.docx]

**Impact of concurrent systemic and inhaled corticosteroids use on clinical outcomes in advanced lung cancer patients receiving immune checkpoint inhibitors**

**Running title: Corticosteroids use during immunotherapy**

Yi Liu^1†^, Jiarui Zhang^1†^, Linhui Yang^1^, Jiadi Gan^1^, Qi Qi^1^, Wanqin Fang^1^, Huohuo Zhang^1^, Rui Xu^1^, Sha Liu^1^, Jun Yang^2^, Weimin Li^1,3,4^, Dan Liu^1,3,4^*

^1^Department of Pulmonary and Critical Care Medicine, West China Hospital, Sichuan University, Chengdu, 610041, Sichuan, China.

^2^Department of Pulmonary and Critical Care Medicine, Langzhong People’s Hospital, Langzhong, 637400, Sichuan, China.

^3^State Key Laboratory of Respiratory Health and Multimorbidity, West China Hospital, Chengdu, 610041, Sichuan, China.

^4^Institute of Respiratory Health, West China Hospital, Sichuan University, Chengdu, 610041, Sichuan, China.

^†^These authors contributed equally to this manuscript.

^*^Corresponding author.

**Correspondence:**

1. Dan Liu, MD

Department of Pulmonary and Critical Care Medicine, West China Hospital, Sichuan University, Chengdu, 610041, Sichuan, China.

State Key Laboratory of Respiratory Health and Multimorbidity, West China Hospital, Chengdu, 610041, Sichuan, China.

Institute of Respiratory Health, West China Hospital, Sichuan University, Chengdu, 610041, Sichuan, China.

Email: [liudan10965@wchscu.cn](mailto:liudan10965@wchscu.cn)

# Abstract

**Background:** Corticosteroids are frequently used during immune checkpoint inhibitor (ICI) treatment, especially in lung cancer patients with comorbidities. Previous studies suggest that systemic corticosteroids (SCS) hinder the effectiveness of ICIs, while the impact of inhaled corticosteroids (ICS) remains unclear. We aimed to examine the association between concurrent SCS and ICS on clinical outcomes in advanced lung cancer patients treated with ICIs.

**Methods:** This retrospective cohort study enrolled adults with advanced lung cancer who initiated ICIs between September 1 2016 and September 30 2023 at West China Hospital of Sichuan University. Exposure included concurrent SCS, ICS versus no steroid treatment. Clinical outcomes including overall survival (OS), progression-free survival (PFS), and tumor response were assessed. Time-dependent Cox regression models (treating SCS and ICS use as time-varying covariates) were applied to account for immortal-time bias.

**Results:** Among 368 patients, 122 were SCS users, 51 were ICS users and 195 did not receive corticosteroids. SCS use was associated with inferior PFS (hazard ratio [HR] 1.99; 95% confidence interval [CI], 1.40-2.84; p value<0.001) and OS (HR 1.77; 95% CI, 1.25-2.51; p value=0.001), whereas ICS use was not significantly associated with PFS (HR 1.35; 95% CI, 0.66-2.80; p value=0.412) or OS (HR 1.48; 95% CI, 0.74-2.97; p value=0.269). Subgroup and sensitivity analyses generally supported the robustness of these findings. In exploratory analyses restricted to SCS users, initiation of SCS within 2 months after ICI start was associated with worse survival.

**Conclusions:** This study suggests that concurrent SCS use may adversely affect the clinical outcomes of advanced lung cancer patients receiving immunotherapy, whereas ICS use did not appear to compromise ICI efficacy. These findings highlight the critical need for cautious consideration when combining ICIs with systemic corticosteroids and emphasize the importance of treating both cancer and lung comorbidity simultaneously.

# Keywords

Systemic corticosteroids; inhaled corticosteroids; immune checkpoint inhibitors; advanced lung cancer; efficacy.

# Introduction

Immune checkpoint inhibitors (ICIs) have transformed the landscape of oncologic treatment [1]. ICIs, represented by programmed cell death ligand 1 (PD-L1) inhibitors, programmed cell death 1 (PD-1) inhibitors, and cytotoxic T-lymphocyte-associated protein 4 (CTLA-4) inhibitors, have become standard therapies [2, 3]. Emerging data has demonstrated their significant clinical advantages in advanced lung cancer patients [4-7]. However, these benefits were identified exclusively in a subset of patients who showed positive response to ICI therapy [8]. Therefore, there has been a pressing need to identify predictors of ICI response and optimize treatment strategies.

Given the prevalence of lung cancer comorbidities, concerns have been raised regarding concomitant medication use during ICI therapy, which may interact with the immune system [9, 10]. Corticosteroids (CS), including systemic and inhaled corticosteroids, play an indispensable role in clinical practice for comprehensive indications, such as managing immune-related adverse events (irAEs), palliating cancer-related symptoms (anorexia, dyspnea, pain, symptomatic brain metastasis, etc), and treating comorbid conditions (chronic obstructive pulmonary disease, rheumatic disease, allergies, etc) [11-13]. Systemic corticosteroids (SCS) have been shown to diminish the ICI effectiveness in advanced lung cancer patients by inducing immunosuppression [11, 14-16]. However, the effect of inhaled corticosteroids (ICS) on clinical outcomes in ICI-treated advanced lung cancer patients remains unclear. Clinical data on ICS in this setting are scarce and mainly derived from retrospective cohorts including heterogeneous advanced cancers. Several studies have reported that baseline inhaled corticosteroid use was not associated with shorter survival in contrast to systemic corticosteroids use, whereas one study observed an association between ICS and an increased risk of checkpoint inhibitor pneumonitis [17-19]. In addition, studies evaluating ICI responses in small-cell lung cancer (SCLC) are limited, as SCLC patients have often been excluded from previous clinical trials. Thus, further studies are needed to elucidate the correlation between CS prescription and clinical outcomes in advanced lung cancer patients.

To address this issue in the literature, this study aimed to examine the association between concomitant corticosteroids (SCS and ICS) and the efficacy of ICIs in advanced lung cancer patients.

# Methods

## Design and study population

This single-center retrospective study was conducted at West China Hospital of Sichuan University, including consecutive patients who initiated ICIs between September 1 2016 and September 30 2023. Eligible participants included adults (age≥18 years) with advanced lung cancer who had been treated with single-agent PD-(L)1 inhibitor (atezolizumab, sintilimab, pembrolizumab, durvalumab, nivolumab, tislelizumab, or camrelizumab) or combined with CTLA-4 inhibitor, ipilimumab. Patients were excluded if they received less than two cycles of PD-(L)1 immunotherapy, and received other concomitant chemotherapy or targeted therapy. Those enrolled in clinical trials potentially receiving placebos were also excluded.

Demographic data and patient characteristics were retrieved from electronic health records. Clinicopathologic characteristics including age, sex, body mass index (BMI), smoking status, comorbidities (recorded based on physician-documented diagnoses in the electronic medical records), ICI use (treatment line, agent used), corticosteroid use (type, indication, route of administration, date of corticosteroid initiation during ICI treatment), histology, site of metastases, PD-L1 tumor proportion score (TPS) (evaluated via immunohistochemistry), Eastern Cooperative Oncology Group Performance Status (ECOG PS) at the start of treatment, death, disease progression, and tumor response evaluation, were collected. All participants were followed until death or data lock (April 1 2024), whichever came first. This study received approval from the institutional ethics committee of West China Hospital, Sichuan University (No. 2020-232). Due to the retrospective nature of the study, informed consent from participants was waived.

## Exposure and response evaluation

Patients’ pharmacy records were reviewed to determine corticosteroid prescriptions. Concurrent corticosteroid use was defined as any prescription of systemic or inhaled corticosteroids during ICI treatment. Exposures included treatment with systemic corticosteroids or inhaled corticosteroids versus no steroid treatment. The administration of SCS at a dose equivalent to ≥10mg of prednisone during immunotherapy was documented, as doses <10mg were generally not included from clinical trials and were considered within the range of physiologic adrenal replacement [20, 21]. Systemic steroids included dexamethasone, methylprednisolone, prednisone, prednisolone, and hydrocortisone. Only SCS administered through oral routes, intravenous, or intramuscular were considered. Systemic corticosteroid indications were classified as cancer-unrelated, cancer-related and irAEs. Cancer-related indications included cancer-related symptoms (dyspnea, symptomatic brain metastasis, cancer-related pain, spinal cord compression, and superior vena cava syndrome) and premedications of chemotherapy. The main cancer-unrelated indications were chemotherapy or radiation pneumonitis, chronic obstructive pulmonary disease (COPD) exacerbation, pulmonary infection, dermatomyositis and endocrine disorders requiring steroid replacement therapy. irAEs included immune-related pneumonitis, myocarditis, nephritis, thyroiditis, enterocolitis, encephalitis, and dermatologic adverse events. Concurrent ICS use was defined as any prescription of inhaled budesonide, fluticasone, and beclomethasone overlapping with ICI treatment. Intranasal corticosteroids were excluded. The indications for ICS included chronic obstructive pulmonary disease or asthma.

The primary outcome was overall survival (OS), defined as the time elapsed between ICI initiation and death of any cause. We also evaluated progression-free survival (PFS), disease control rate (DCR), and objective response rate (ORR). PFS was defined as the time interval from ICI initiation to the earliest occurrence of disease progression or death. DCR was defined as the sum of complete response (CR), partial response (PR), and stable disease (SD), while ORR was defined as CR plus PR. Participants who remained alive and did not experience disease progression were censored at the end of the study (April 1, 2024). Response rates were determined by independent radiologists utilizing the Response Evaluation Criteria in Solid Tumors (RECIST 1.1) [22].

## Statistical analysis

Baseline demographic and characteristics were described as number (percentage) for categorical variables, and median (interquartile range) for continuous variables. Intergroup comparisons were conducted using one-way analysis of variance or the Kruskal-Wallis test for quantitative data, and Chi-square test or Fisher’s exact test for qualitative variables. Missing data were handled using multiple imputation. To account for potential immortal-time bias, time-dependent Cox regression was applied to estimate hazard ratios (HRs) and 95% confidence intervals (CIs) for PFS and OS, treating corticosteroid exposure (both SCS and ICS) as a time-varying covariate. Variables that were univariately associated with outcomes (P<0.10) were incorporated into the multivariate analysis. Proportional hazards assumptions were assessed using Schoenfeld residuals. Cox regression model was employed as a supplementary contrast analysis. Survival curves were estimated by the Kaplan-Meier method, with group comparisons conducted using the log-rank test. Statistical tests were conducted at a significance threshold of p<0.05 (two-tailed). All analyses were conducted utilizing R software version 4.5.1 and SPSS version 26.0.

# Results

## Patient characteristics

Among the 4,064 consecutive patients who received PD-(L)1 inhibitor for advanced lung cancer between September 2016 and September 2023, we finally identified 368 participants who met the study criteria. Of 368 patients, 122 received concurrent systemic steroids, 51 received inhaled corticosteroids, and 195 did not receive steroid treatment (Figure 1). A summary of patients’ demographic and characteristics is presented in Table 1. The median age was 66 years (interquartile range [IQR], 58.0 to 71.1 years), with most of patients being male (87.0%), ever smokers (64.9%) and having a baseline ECOG PS of 0-1 (89.7%). Adenocarcinoma (48.4%) was the most frequent histological subtype, followed by squamous cell carcinoma (41.8%), small cell carcinoma (5.2%) and other subtypes (4.6%). ICIs were administered to 46.7% of cases as first line therapy. 81.5% received PD-1 inhibitors and the rest received anti-PD-L1 agents. Bone metastases were present in 23.9% of the participants, brain metastases in 16.8% and liver metastases in 10.9%. Patients’ characteristics, in terms of sex, smoking status, ICI therapy and PD-L1 TPS, were generally well balanced across groups. As excepted, concurrent steroid users were older and were more likely to have poor ECOG PS and more respiratory diseases.

Regarding corticosteroid indications, 79 patients (64.8%) received concurrent SCS for cancer-related indications, 16 (13.1%) for cancer-unrelated indications, and 27 (22.1%) for irAEs. 49 patients (96.1%) received concurrent ICS for COPD and 2 (3.9%) for asthma. The timing and detailed indication for SCS administration are depicted in Table S1. Overall, 47 patients (38.5%) initiated SCS within 2 months after ICI initiation, whereas 75 (61.5%) started SCS ≥2 months after ICI initiation. The most common cancer-related indications were cancer-related symptoms (42.7%), and the main cancer-unrelated indications were chemotherapy or radiation pneumonitis (5.0%). Immune-related pneumonitis represented the most frequent irAE-related indication (11.5%). Dexamethasone was the most frequently used steroid (54.9%), followed by methylprednisolone and prednisone.

## Clinical outcomes

The median follow-up duration was 21.0 months in the entire cohort. Kaplan-Meier analysis showed significant differences in PFS across the three groups (log-rank p value=0.002; Figure 2A). The median PFS was 11.0 months in the SCS group, 17.0 months in the no steroids group, and 25.0 months in the ICS group. For overall survival, the median OS was 21.0 months in the SCS group, 35.0 months in the no steroids group, and 21.0 months in the ICS group (log-rank p value=0.002; Figure 2B). The ORR was 16.0%, while DCR was 91.0% in the total population. Significant differences regarding ORR (19.0% vs 9.0% vs 21.6%; p value=0.032; Figure 2C) and DCR (93.3% vs 85.2% vs 96.1%; p value=0.020; Figure 2D) were also detected between groups.

Time-dependent Cox regression results for PFS and OS are presented in Table 2. After adjusting for age, BMI, smoking status, ICI agent, PD-L1 TPS, comorbidities, laboratory parameters, and history of liver and bone metastasis, multivariate analysis indicated that SCS use was associated with inferior PFS (hazard ratio [HR] 1.99; 95% confidence interval [CI], 1.40-2.84; p value<0.001) and OS (HR 1.77; 95% CI, 1.25-2.51; p value=0.001). Whereas, ICS use did not show a statistically significant association with PFS (HR 1.35; 95% CI, 0.66-2.80; p value=0.412) or OS (HR 1.48; 95% CI, 0.74-2.97; p value=0.269). Cardiovascular diseases, bone metastasis and liver metastasis were significantly associated with an increased risk of disease progression or death. In contrast, PD-L1 positivity was found to be correlated with significantly improved PFS and OS. Multivariate Cox regression analysis yielded consistent results, which indicated that SCS use was linked to worse survival outcomes. ICS use remained not significantly associated with PFS and but showed a statistically significant association with improved OS (HR 0.57; 95% CI, 0.35-0.93; p value=0.024) (Table S2). This discrepancy may reflect residual immortal-time bias. Nevertheless, the general consistency between models reinforces the stability of the findings. Additional sensitivity analysis indicated that ICI agent did not change the associations between SCS or ICS exposure after multivariable adjustment (Table S3).

## Subgroup analysis

We further examined the association in populations with different characteristics, using multivariable time-dependent Cox models with SCS and ICS included as time-varying covariates (Figure 3). Subgroup analyses for overall survival demonstrated the robustness of clinical outcomes in general. The detrimental association between SCS exposure and OS was generally observed across all subgroups. In contrast, ICS exposure did not show a clear or consistent association with OS, reflecting the limited number of ICS users within each subgroup. In an exploratory analysis restricted to patients receiving concurrent SCS, we examined the impact of SCS timing, type and indication on ICI efficacy. Compared with SCS initiation≥2 months, <2 months was linked to decreased overall survival (median OS 11.0 vs 24.0 months; p value=0.024; Figure S1A), whereas PFS did not differ significantly between the two groups (median PFS 8.0 vs 12.0 months; p value=0.669; Figure S1D). SCS indications showed no significant difference in OS (median OS 22.0 vs 21.0 months vs 13.0 months; p value=0.930; Figure S1B) and PFS (median PFS 11.0 vs 11.0 months vs 9.0 months; p value=0.954; Figure S1E). Additionally, OS (median OS 17.0 vs 22.0 months; p value=0.824; Figure S1C) and PFS (median PFS 11.0 vs 10.0 months; p value=0.495; Figure S1F) were comparable between patients receiving short- or intermediate-acting versus long-acting SCS. Patients received dexamethasone had poor survival compared to the steroids-naïve population (HR 1.43; 95% CI, 1.01-2.04; p value=0.044; Figure S2). The sensitivity analyses restricted to COPD patients (Table S4) and non-small cell lung cancer patients (Table S5) yielded results consistent with those of the overall cohort, confirming the robustness of our findings.

# Discussion

Immune checkpoint inhibitors are currently important anti-cancer approaches. Although ICIs have demonstrated remarkable effectiveness in lung cancer treatment, not all patients exhibit favorable responses [23, 24]. Therefore, the identification of predictors of ICI response is desperately needed. In this retrospective study, we conducted time-dependent Cox analysis to address the potential immortal-time bias. After adjusting for clinically relevant variables, SCS exposure was consistently associated with inferior PFS and OS, as well as a lower tumor response rate, indicating that SCS may attenuate the therapeutic benefit of ICIs. In contrast, ICS prescription did not adversely affect survival outcomes, even in patients with COPD who frequently require ICS therapy. These associations were robust to challenge in subgroup analyses. In exploratory analyses restricted to SCS users, early SCS initiation (<2 months after ICI start) was associated with shorter OS, whereas SCS indication and SCS type were not significantly related to PFS or OS.

Given the prevalence of lung cancer comorbidities, there are growing public concerns over the use of concomitant medication during ICI treatment which may interfere with the immune system [9, 25, 26]. Systemic corticosteroids are widely used and play an essential role in oncologic treatment for various indications. It has raised the concern that SCS may theoretically undermine the efficacy of immunotherapy due to their immunosuppressive properties, such as impairing T-lymphocytes proliferation and differenciation [27, 28]. Several studies have consistently shown the adverse effect of systemic steroids on survival in ICI-treated lung cancer patients [14, 29-31]. A recent meta-analysis indicated that corticosteroids negatively affect both mortality and progression in patients with non-small cell lung cancer (NSCLC) receiving ICIs [11]. After adjusting for potential confounders, our time-dependent Cox analysis of advanced lung cancer patients indicated that SCS exposure was associated with worse survival outcomes, which was in accordance with these findings. The supplementary Cox regression further confirmed the negative effect of SCS, supporting the robustness of this association despite different model specifications. To further address potential heterogeneity caused by inclusion of SCLC cases, we performed a sensitivity analysis restricted to NSCLC patients, and the results were consistent with those of the overall cohort, supporting the robustness of our conclusions. Consequently, the use of systemic corticosteroids should be considered cautiously during immunotherapy.

However, not all SCS use is detrimental. Previous studies have reported that SCS administration for irAEs and cancer-unrelated symptoms did not correlate with lower OS in NSCLC patients undergoing immunotherapy [11, 14, 15]. To further refine the impact of SCS, we explored the timing, types and indications of SCS prescription. Align with prior retrospective studies [32], we found that patients who initiated SCS ＜2 months after ICI start had shorter OS, whereas PFS did not differ significantly between early and later SCS initiation. By contrast, SCS indication and SCS type were not significantly associated with OS or PFS in our cohort. These findings should be interpreted with caution, as early SCS use likely reflects more aggressive disease, higher symptom burden and poorer baseline prognosis, and confounding by indication cannot be excluded. Our research provides insights into the negative effect of concurrent medication use on clinical outcomes in patients receiving immunotherapy. On the basis of these findings, it may be prudent to delay SCS initiation and explore alternative pharmacologic strategies to treat cancer-related indications in patients receiving ICI treatment.

As essential medicines listed by the World Health Organization, inhaled corticosteroids are among the most prescribed medications worldwide. Although previous studies have determined that systemic corticosteroids negatively affected the prognosis of lung cancer patients receiving ICIs, evidence regarding the association between ICS and ICI efficacy remains limited. In contrast to existing literature, we found that inhaled corticosteroids administration did not appear to influence survival or tumor response rate in advanced lung cancer patients treated with ICIs, suggesting that ICS may be a viable alternative for patients undergoing ICIs in certain cases. While the traditional Cox model indicated a survival benefit associated with ICS, this association disappeared after applying a time-dependent Cox model. The attenuation of statistical significance suggests that the apparent OS benefit observed in the conventional Cox analysis may be partly attributable to residual immortal-time bias. One possible explanation for this discrepancy is that ICS was predominantly prescribed to patients with COPD, a population that has consistently shown improved responses to ICI therapy. Prior studies have shown that COPD could alter the immune cell composition and enhance the efficacy of ICIs by modulating the tumor microenvironment [33-35]. Additionally, inhaled corticosteroids are known to exert potent anti-inflammatory effects in the airways by binding to the glucocorticoid receptor and repressing NF-κB and activator protein-1-driven transcription of multiple inflammatory genes, leading to reduced production of pro-inflammatory cytokines and chemokines such as IL-8 and TNF-α and attenuation of neutrophilic airway inflammation. In patients with COPD, ICS therapy has been shown to decrease airway inflammatory burden, including reductions in sputum neutrophils and neutrophil-associated mediators, and to improve markers of airway inflammation [36]. By dampening chronic airway inflammation and epithelial injury in the bronchial tree while producing relatively low systemic glucocorticoid exposure, ICS could theoretically help maintain a more favorable pulmonary immune milieu during PD-(L)1 blockade. In our cohort, ICS use might not have a detrimental effect on PFS and OS, which is in line with previous retrospective studies [17, 18]. Prospective studies with detailed characterization of airway disease, ICS exposure and immune-related toxicity are warranted. Furthermore, ICS can reduce the frequency of exacerbations in comorbid conditions such as asthma and COPD, maintaining better lung function and enhancing patient tolerance to treatment. In our study, standardized ICS protocols were administered to patients with COPD in accordance with clinical guidelines. To further address COPD-related confounding, we conducted a sensitivity analysis restricted to COPD patients. The time-dependent Cox analysis demonstrated ICS use did not compromise the efficacy of ICIs in the COPD subgroup. Therefore, our findings add to the evidence base that ICS does not adversely affect ICI efficacy, and emphasize the necessity of simultaneously managing both cancer and lung among ICI-treated individuals with advanced lung cancer.

To our knowledge, this study is the first to evaluate the impact of both systemic and inhaled corticosteroids on the efficacy of ICIs among patients with advanced lung cancer in China. Our findings provide valuable insights into the concurrent corticosteroids alongside ICI treatment, which can inform the decision-making in clinical practice.

Several limitations of this study need to be acknowledged. Firstly, this retrospective single-center study may be subject to selection bias and unmeasured confounding that may not be fully mitigated despite multivariable adjustment. Therefore, further study with a larger sample size and a prospective design is needed to validate these findings. Secondly, our study concentrated on patients who received PD-(L)1 inhibitors monotherapy, and those treated with fewer than two cycles or in combination with chemotherapy or targeted therapy were excluded. This limits the generalizability of our findings to broader populations. Thirdly, detailed information regarding the cumulative dose, tapering patterns, and duration of systemic corticosteroid therapy could not be reliably quantified because these data were documented inconsistently in real-world clinical practice, with frequent intermittent or overlapping prescriptions. Therefore, corticosteroid exposure was analyzed based on the timing of initiation relative to ICI treatment, which was the most consistently recorded and clinically interpretable measure available. Fourth, our cohort included both NSCLC and SCLC, which differ in clinical behavior and underlying biology, but the limited number of SCLC cases precluded robust histology-specific analyses. Thus, our findings should be interpreted primarily in the context of advanced NSCLC and extrapolated to SCLC with caution. In addition, irAEs were not systematically captured, and data on the exact timing, organ involvement and grade were incomplete. Consequently, we were unable to robustly evaluate their impact on ICI efficacy. Moreover, information on tumor mutational burden and lung function test data were not consistently available. Therefore, COPD severity could not be uniformly classified, and residual confounding related to COPD severity cannot be excluded. Ultimately, although standardized prescription criteria and a sensitivity analysis adjusting for ICI agent yielded results consistent with the main analyses, heterogeneity of immunotherapy strategies and residual confounding related to ICI type cannot be entirely excluded.

# Conclusions

In summary, this retrospective study demonstrated that concurrent systemic corticosteroids use were significantly associated with inferior PFS, OS and tumor response in advanced lung cancer patients treated with ICIs. Whereas the prescription of inhaled corticosteroids showed no detrimental impact on ICI efficacy, even in patients with COPD who frequently require ICS therapy. These findings underscore the importance of careful consideration when administering systemic corticosteroids during ICI treatment and emphasize the necessity for treating both cancer and lung simultaneously. Further studies involving larger sample size are warranted to validate and expand these findings to other populations.

# Abbreviations

BMI, body mass index; CTLA-4, cytotoxic T-lymphocyte-associated protein 4; CS, corticosteroids; COPD, chronic obstructive pulmonary disease; CKD, chronic kidney disease; CR, complete response; CI, confidence interval; DCR, disease control rate; ECOG PS, Eastern Cooperative Oncology Group Performance Status; HR, hazard ratio; ICI, immune checkpoint inhibitor; ICS, inhaled corticosteroids; IQR, interquartile range; irAE, immune-related adverse event; LDH, lactate dehydrogenase; NSCLC, non-small cell lung cancer; NLR, neutrophil-to-lymphocyte ratio; OS, overall survival; ORR, objective response rate; PFS, progression-free survival; PLR, platelet-to-lymphocyte ratio; PR, partial response; PD, progressive disease; PD-1, programmed cell death 1; PD-L1, programmed cell death ligand 1; RECIST, Response Evaluation Criteria in Solid Tumors; SCS, systemic corticosteroids; SD, stable disease; SCLC, small-cell lung cancer; TPS, tumor proportion score.

# Declarations

# **Author contributions**

Study design: Yi Liu and Dan Liu. Data acquisition: Yi Liu, Jiarui Zhang, Linhui Yang, Jiadi Gan, Qi Qi, Wanqin Fang, Huohuo Zhang, Rui Xu, Sha Liu, and Jun Yang. Data analysis, interpretation, and manuscript writing: Yi Liu. Revision and editing of manuscript: Yi Liu, Jiarui Zhang, Weimin Li and Dan Liu. All authors had final consent to submit the manuscript for publication.

# **Funding**

This study was supported by the National Natural Science Foundation of China (82173182), the Chengdu Science and Technology Project (2023-YF09-00039-SN) and the Science and Technology Program of Sichuan (2023NSFSC1939).

# **Data availability statement**

Research data can be accessed upon justified request by contacting the corresponding author.

# **Ethics statement**

Ethics approval was provided by the ethics committee of West China Hospital, Sichuan University (No. 2020-232). The informed consent from patients was waived due to the retrospective cohort study design.

# **Consent for publication**

Not applicable.

# **Conflict of interest**

All authors declare that there are no competing interests pertain to this study.

# References

1. Robert C: A decade of immune-checkpoint inhibitors in cancer therapy. Nat Commun 2020, 11:3801.

2. Jaiyesimi IA, Leighl NB, Ismaila N, Alluri K, Florez N, Gadgeel S, Masters G, Schenk EL, Schneider BJ, Sequist L, et al: Therapy for Stage IV Non-Small Cell Lung Cancer Without Driver Alterations: ASCO Living Guideline, Version 2023.3. J Clin Oncol 2024, 42:e23-e43.

3. Hendriks LE, Kerr KM, Menis J, Mok TS, Nestle U, Passaro A, Peters S, Planchard D, Smit EF, Solomon BJ, et al: Non-oncogene-addicted metastatic non-small-cell lung cancer: ESMO Clinical Practice Guideline for diagnosis, treatment and follow-up. Ann Oncol 2023, 34:358-376.

4. Cho BC, Abreu DR, Hussein M, Cobo M, Patel AJ, Secen N, Lee KH, Massuti B, Hiret S, Yang JCH, et al: Tiragolumab plus atezolizumab versus placebo plus atezolizumab as a first-line treatment for PD-L1-selected non-small-cell lung cancer (CITYSCAPE): primary and follow-up analyses of a randomised, double-blind, phase 2 study. Lancet Oncol 2022, 23:781-792.

5. Wang L, Luo Y, Ren S, Zhang Z, Xiong A, Su C, Zhou J, Yu X, Hu Y, Zhang X, et al: A Phase 1b Study of Ivonescimab, a Programmed Cell Death Protein-1 and Vascular Endothelial Growth Factor Bispecific Antibody, as First- or Second-Line Therapy for Advanced or Metastatic Immunotherapy-Naive NSCLC. J Thorac Oncol 2024, 19:465-475.

6. Zhao Y, Ma Y, Fan Y, Zhou J, Yang N, Yu Q, Zhuang W, Song W, Wang ZM, Li B, et al: A multicenter, open-label phase Ib/II study of cadonilimab (anti PD-1 and CTLA-4 bispecific antibody) monotherapy in previously treated advanced non-small-cell lung cancer (AK104-202 study). Lung Cancer 2023, 184:107355.

7. Ferrara R, Imbimbo M, Malouf R, Paget-Bailly S, Calais F, Marchal C, Westeel V: Single or combined immune checkpoint inhibitors compared to first-line platinum-based chemotherapy with or without bevacizumab for people with advanced non-small cell lung cancer. Cochrane Database Syst Rev 2021, 4:Cd013257.

8. Larkin J, Chiarion-Sileni V, Gonzalez R, Grob JJ, Rutkowski P, Lao CD, Cowey CL, Schadendorf D, Wagstaff J, Dummer R, et al: Five-Year Survival with Combined Nivolumab and Ipilimumab in Advanced Melanoma. N Engl J Med 2019, 381:1535-1546.

9. Hong S, Lee JH, Heo JY, Suh KJ, Kim SH, Kim YJ, Kim JH: Impact of concurrent medications on clinical outcomes of cancer patients treated with immune checkpoint inhibitors: analysis of Health Insurance Review and Assessment data. J Cancer Res Clin Oncol 2024, 150:186.

10. Buti S, Bersanelli M, Perrone F, Tiseo M, Tucci M, Adamo V, Stucci LS, Russo A, Tanda ET, Spagnolo F, et al: Effect of concomitant medications with immune-modulatory properties on the outcomes of patients with advanced cancer treated with immune checkpoint inhibitors: development and validation of a novel prognostic index. Eur J Cancer 2021, 142:18-28.

11. Li N, Zheng X, Gan J, Zhuo T, Li X, Yang C, Wu Y, Qin S: Effects of glucocorticoid use on survival of advanced non-small-cell lung cancer patients treated with immune checkpoint inhibitors. Chin Med J (Engl) 2023, 136:2562-2572.

12. Hui D, Puac V, Shelal Z, Dev R, Hanneman SK, Jennings K, Ma H, Urbauer DL, Shete S, Fossella F, et al: Effect of dexamethasone on dyspnoea in patients with cancer (ABCD): a parallel-group, double-blind, randomised, controlled trial. Lancet Oncol 2022, 23:1321-1331.

13. Schneider BJ, Naidoo J, Santomasso BD, Lacchetti C, Adkins S, Anadkat M, Atkins MB, Brassil KJ, Caterino JM, Chau I, et al: Management of Immune-Related Adverse Events in Patients Treated With Immune Checkpoint Inhibitor Therapy: ASCO Guideline Update. J Clin Oncol 2021, 39:4073-4126.

14. Ricciuti B, Dahlberg SE, Adeni A, Sholl LM, Nishino M, Awad MM: Immune Checkpoint Inhibitor Outcomes for Patients With Non-Small-Cell Lung Cancer Receiving Baseline Corticosteroids for Palliative Versus Nonpalliative Indications. J Clin Oncol 2019, 37:1927-1934.

15. Skribek M, Rounis K, Afshar S, Grundberg O, Friesland S, Tsakonas G, Ekman S, De Petris L: Effect of corticosteroids on the outcome of patients with advanced non-small cell lung cancer treated with immune-checkpoint inhibitors. Eur J Cancer 2021, 145:245-254.

16. Goodman RS, Johnson DB, Balko JM: Corticosteroids and Cancer Immunotherapy. Clin Cancer Res 2023, 29:2580-2587.

17. Kostine M, Mauric E, Tison A, Barnetche T, Barre A, Nikolski M, Rouxel L, Dutriaux C, Dousset L, Prey S, et al: Baseline co-medications may alter the anti-tumoural effect of checkpoint inhibitors as well as the risk of immune-related adverse events. Eur J Cancer 2021, 157:474-484.

18. Gaucher L, Adda L, Sejourne A, Joachim C, Chaby G, Poulet C, Liabeuf S, Gras-Champel V, Masmoudi K, Moreira A, et al: Impact of the corticosteroid indication and administration route on overall survival and the tumor response after immune checkpoint inhibitor initiation. Ther Adv Med Oncol 2021, 13:1758835921996656.

19. Li M, Spakowicz D, Zhao S, Patel SH, Johns A, Grogan M, Miah A, Husain M, He K, Bertino EM, et al: Brief report: inhaled corticosteroid use and the risk of checkpoint inhibitor pneumonitis in patients with advanced cancer. Cancer Immunol Immunother 2020, 69:2403-2408.

20. Hellmann MD, Paz-Ares L, Bernabe Caro R, Zurawski B, Kim SW, Carcereny Costa E, Park K, Alexandru A, Lupinacci L, de la Mora Jimenez E, et al: Nivolumab plus Ipilimumab in Advanced Non-Small-Cell Lung Cancer. N Engl J Med 2019, 381:2020-2031.

21. Paz-Ares L, Ciuleanu TE, Cobo M, Schenker M, Zurawski B, Menezes J, Richardet E, Bennouna J, Felip E, Juan-Vidal O, et al: First-line nivolumab plus ipilimumab combined with two cycles of chemotherapy in patients with non-small-cell lung cancer (CheckMate 9LA): an international, randomised, open-label, phase 3 trial. Lancet Oncol 2021, 22:198-211.

22. Eisenhauer EA, Therasse P, Bogaerts J, Schwartz LH, Sargent D, Ford R, Dancey J, Arbuck S, Gwyther S, Mooney M, et al: New response evaluation criteria in solid tumours: revised RECIST guideline (version 1.1). Eur J Cancer 2009, 45:228-247.

23. Wagner G, Stollenwerk HK, Klerings I, Pecherstorfer M, Gartlehner G, Singer J: Efficacy and safety of immune checkpoint inhibitors in patients with advanced non-small cell lung cancer (NSCLC): a systematic literature review. Oncoimmunology 2020, 9:1774314.

24. Hopkins AM, Rowland A, Kichenadasse G, Wiese MD, Gurney H, McKinnon RA, Karapetis CS, Sorich MJ: Predicting response and toxicity to immune checkpoint inhibitors using routinely available blood and clinical markers. Br J Cancer 2017, 117:913-920.

25. Cortellini A, Tucci M, Adamo V, Stucci LS, Russo A, Tanda ET, Spagnolo F, Rastelli F, Bisonni R, Santini D, et al: Integrated analysis of concomitant medications and oncological outcomes from PD-1/PD-L1 checkpoint inhibitors in clinical practice. J Immunother Cancer 2020, 8.

26. Lurienne L, Cervesi J, Duhalde L, de Gunzburg J, Andremont A, Zalcman G, Buffet R, Bandinelli PA: NSCLC Immunotherapy Efficacy and Antibiotic Use: A Systematic Review and Meta-Analysis. J Thorac Oncol 2020, 15:1147-1159.

27. Giles AJ, Hutchinson MND, Sonnemann HM, Jung J, Fecci PE, Ratnam NM, Zhang W, Song H, Bailey R, Davis D, et al: Dexamethasone-induced immunosuppression: mechanisms and implications for immunotherapy. J Immunother Cancer 2018, 6:51.

28. Libert C, Dejager L: How steroids steer T cells. Cell Rep 2014, 7:938-939.

29. Arbour KC, Mezquita L, Long N, Rizvi H, Auclin E, Ni A, Martínez-Bernal G, Ferrara R, Lai WV, Hendriks LEL, et al: Impact of Baseline Steroids on Efficacy of Programmed Cell Death-1 and Programmed Death-Ligand 1 Blockade in Patients With Non-Small-Cell Lung Cancer. J Clin Oncol 2018, 36:2872-2878.

30. Scott SC, Pennell NA: Early Use of Systemic Corticosteroids in Patients with Advanced NSCLC Treated with Nivolumab. J Thorac Oncol 2018, 13:1771-1775.

31. Hendriks LEL, Henon C, Auclin E, Mezquita L, Ferrara R, Audigier-Valette C, Mazieres J, Lefebvre C, Rabeau A, Le Moulec S, et al: Outcome of Patients with Non-Small Cell Lung Cancer and Brain Metastases Treated with Checkpoint Inhibitors. J Thorac Oncol 2019, 14:1244-1254.

32. Maslov DV, Tawagi K, Kc M, Simenson V, Yuan H, Parent C, Bamnolker A, Goel R, Blake Z, Matrana MR, Johnson DH: Timing of steroid initiation and response rates to immune checkpoint inhibitors in metastatic cancer. J Immunother Cancer 2021, 9.

33. Mark NM, Kargl J, Busch SE, Yang GHY, Metz HE, Zhang H, Hubbard JJ, Pipavath SNJ, Madtes DK, Houghton AM: Chronic Obstructive Pulmonary Disease Alters Immune Cell Composition and Immune Checkpoint Inhibitor Efficacy in Non-Small Cell Lung Cancer. Am J Respir Crit Care Med 2018, 197:325-336.

34. Shin SH, Park HY, Im Y, Jung HA, Sun JM, Ahn JS, Ahn MJ, Park K, Lee HY, Lee SH: Improved treatment outcome of pembrolizumab in patients with nonsmall cell lung cancer and chronic obstructive pulmonary disease. Int J Cancer 2019, 145:2433-2439.

35. Zhou J, Chao Y, Yao D, Ding N, Li J, Gao L, Zhang Y, Xu X, Zhou J, Halmos B, et al: Impact of chronic obstructive pulmonary disease on immune checkpoint inhibitor efficacy in advanced lung cancer and the potential prognostic factors. Transl Lung Cancer Res 2021, 10:2148-2162.

36. Lea S, Higham A, Beech A, Singh D: How inhaled corticosteroids target inflammation in COPD. Eur Respir Rev 2023, 32.

**Table 1 Demographic and characteristic of the study population.**

| **Characteristic** | **Overall**  **(n=368)** | **No Steroids**  **(n=195)** | **Concurrent SCS**  **(n=122)** | **Concurrent ICS**  **(n=51)** | ***P value*** |
| --- | --- | --- | --- | --- | --- |
| Age, years | 66.0 (58.0, 71.1) | 65.0 (57.0, 70.0) | 64.3 (57.0, 71.0) | 70.2 (64.7, 75.0) | <0.001 |
| Sex, n (%) |  |  |  |  |  |
| Male | 320 (87.0) | 164 (84.1) | 107 (87.7) | 49 (96.1) | 0.074 |
| Female | 48 (13.0) | 31 (15.9) | 15 (12.3) | 2 (3.9) |  |
| BMI, kg/m^2^ | 22.7 (20.5, 25.0) | 22.6 (20.5, 25.0) | 22.7 (20.3, 24.5) | 23.0 (22.0, 25.0) | 0.174 |
| Smoking status, n (%) |  |  |  |  |  |
| Never | 129 (35.1) | 73 (37.4) | 41 (33.6) | 15 (29.4) | 0.519 |
| Ever | 239 (64.9) | 122 (62.6) | 81 (66.4) | 36 (70.6) |  |
| ECOG PS, n (%) |  |  |  |  |  |
| 0-1 | 330 (89.7) | 183 (93.8) | 105 (86.1) | 42 (82.4) | 0.016 |
| ≥2 | 38 (10.3) | 12 (6.2) | 17 (13.9) | 19 (17.6) |  |
| Histology, n (%) |  |  |  |  |  |
| Adenocarcinoma | 178 (48.4) | 93 (47.7) | 66 (54.1) | 19 (37.3) | 0.007 |
| Squamous | 154 (41.8) | 87 (44.6) | 37 (30.3) | 30 (58.8) |  |
| Small cell | 19 (5.2) | 7 (3.6) | 10 (8.2) | 2 (3.9) |  |
| Other^a^ | 17 (4.6) | 8 (4.1) | 9 (7.4) | 0 (0.0) |  |
| PD-L1 TPS, n (%) |  |  |  |  |  |
| Negative (<1%) | 52 (14.1) | 32 (16.4) | 18 (14.8) | 2 (3.9) | 0.072 |
| Positive (≥1%) | 316 (85.9) | 163 (83.6) | 104 (85.2) | 49 (96.1) |  |
| ICI line, n (%) |  |  |  |  |  |
| 1st line | 172 (46.7) | 88 (45.1) | 59 (48.4) | 25(49.0) | 0.803 |
| ≥2nd line | 196 (53.3) | 107 (54.9) | 63 (51.6) | 26 (51.0) |  |
| ICI therapy, n (%) |  |  |  |  |  |
| Anti-PD-1 antibody | 300 (81.5) | 160 (82.1) | 100 (82.0) | 40 (78.4) | 0.829 |
| Anti-PD-L1 antibody | 68 (18.5) | 35 (17.9) | 22 (18.0) | 11 (21.6) |  |
| Brain metastasis, n (%) |  |  |  |  |  |
| Yes | 62 (16.8) | 36 (18.5) | 24 (19.7) | 2 (3.9) | 0.028 |
| No | 306 (83.2) | 159 (81.5) | 98 (80.3) | 49 (96.1) |  |
| Liver metastasis, n (%) |  |  |  |  |  |
| Yes | 40 (10.9) | 23 (11.8) | 12 (9.8) | 5 (9.8) | 0.833 |
| No | 328 (89.1) | 172 (88.2) | 110 (90.2) | 46 (90.2) |  |
| Bone metastasis, n (%) |  |  |  |  |  |
| Yes | 88 (23.9) | 48 (24.6) | 32 (26.2) | 8 (15.7) | 0.315 |
| No | 280 (76.1) | 147 (75.4) | 90 (73.8) | 43 (84.3) |  |
| Comorbidities, n (%) |  |  |  |  |  |
| Hypertension | 97 (26.4) | 48 (24.6) | 29 (23.8) | 20 (39.2) | 0.079 |
| COPD | 83 (22.6) | 18 (9.2) | 20 (16.4) | 45 (88.2) | <0.001 |
| Diabetes | 57 (15.5) | 30 (15.4) | 21 (17.2) | 6 (11.8) | 0.664 |
| Cardiovascular diseases | 43 (11.7) | 24 (12.3) | 11 (9.0) | 8 (15.7) | 0.426 |
| CKD | 11 (3.0) | 6 (3.1) | 3 (2.5) | 2 (3.9) | 0.871 |
| NLR | 3.4 (2.3, 5.0) | 3.2 (2.1, 5.1) | 3.5 (2.3, 5.4) | 3.6 (2.5, 4.8) | 0.252 |
| PLR | 162.7 (113.3, 257.9) | 155.7 (107.8, 243.9) | 185.7 (117.5, 308.6) | 155.0 (115.9, 211.7) | 0.083 |
| LDH, UI/L | 191.0 (162.0, 211.0) | 190.0 (157.0, 207.0) | 197.9 (166.0, 225.0) | 179.0 (157.0, 200.0) | 0.073 |
| Corticosteroid indication, n (%) |  |  |  |  |  |
| Cancer-related^b^ | - | - | 79 (64.8) | - |  |
| Cancer-unrelated^c^ | - | - | 16 (13.1) | - |  |
| irAEs | - | - | 27 (22.1) | - |  |
| COPD | - | - | - | 49 (96.1) |  |
| Asthma | - | - | - | 2 (3.9) |  |
| Data are given as n (%) or median (IQR). Significant at p < 0.05.  Abbreviations: IQR, interquartile range; SCS, systemic corticosteroids; ICS, inhaled corticosteroids; BMI, body mass index; ECOG PS, Eastern Cooperative Oncology Group Performance Status; PD-L1, programmed cell death ligand 1; TPS, tumor proportion score; ICI, immune checkpoint inhibitor; PD-1, programmed cell death 1; COPD, chronic obstructive pulmonary disease; CKD, chronic kidney disease; NLR, neutrophil-to-lymphocyte ratio; PLR, platelet-to-lymphocyte ratio; LDH, lactate dehydrogenase; irAE, immune-related adverse event.  ^a^Other histologic types include sarcomatoid carcinoma, pleomorphic carcinoma, lymphoepithelioma-like carcinoma and large cell neuroendocrine carcinoma.  ^b^Cancer-related indications include cancer-related symptoms and premedication for chemotherapy. Cancer-related symptoms include dyspnea, symptomatic brain metastasis, cancer-related pain, spinal cord compression and superior vena cava syndrome.  ^c^The main cancer-unrelated indications were chemotherapy or radiation pneumonitis, COPD exacerbation, pulmonary infection, dermatomyositis and endocrine disorders requiring steroid replacement therapy. | | | | | |

# Table 2 Univariate and multivariate time-dependent Cox regression analyses for PFS and OS of the study population.

| **Factor** | | **Category** | **PFS** | | | | **OS** | | | |
| --- | --- | --- | --- | --- | --- | --- | --- | --- | --- | --- |
|  |  |  | **Univariate analysis** | | **Multivariate analysis** | | **Univariate analysis** | | **Multivariate analysis** | |
|  |  |  | HR (95% CI) | *P* | HR (95% CI) | *P* | HR (95% CI) | *P* | HR (95% CI) | *P* |
| Age | | ≥65 y vs. <65 y | 1.02 (0.80-1.31) | 0.865 |  |  | 1.32 (0.99-1.75) | 0.057 | 1.20 (0.88-1.64) | 0.252 |
| Sex | | Male vs. female | 0.78 (0.55-1.10) | 0.161 |  |  | 1.12 (0.74-1.70) | 0.584 |  |  |
| BMI | | ≥24 vs. <24 | 0.75 (0.58-0.98) | 0.037 | 0.84 (0.63-1.10) | 0.206 | 0.53 (0.39-0.73) | <0.001 | 0.56 (0.40-0.78) | 0.001 |
| Smoking status | | Ever vs. never | 0.96 (0.74-1.25) | 0.775 |  |  | 1.29 (0.96-1.74) | 0.092 | 1.36 (1.00-1.87) | 0.051 |
| ECOG PS | | ≥2 vs. 0-1 | 1.01 (0.68-1.51) | 0.944 |  |  | 1.02 (0.65-1.60) | 0.943 |  |  |
| Squamous | | Yes vs. no | 0.93 (0.72-1.19) | 0.559 |  |  | 1.07 (0.81-1.43) | 0.617 |  |  |
| PD-L1 positive^*^ | | Yes vs. no | 0.60 (0.43-0.83) | 0.002 | 0.58 (0.42-0.80) | 0.001 | 0.69 (0.47-0.99) | 0.043 | 0.57 (0.39-0.84) | 0.004 |
| ICI line | | 1st vs ≥2nd | 0.95 (0.74-1.22) | 0.700 |  |  | 1.03 (0.78-1.36) | 0.860 |  |  |
| ICI therapy | | PD-1 vs. PD-L1 | 1.30 (0.91-1.86) | 0.152 |  |  | 1.84 (1.16-2.93) | 0.010 | 1.42 (0.88-2.29) | 0.152 |
| Brain metastasis | | Yes vs. no | 1.08 (0.78-1.49) | 0.662 |  |  | 1.20 (0.84-1.71) | 0.305 |  |  |
| Liver metastasis | | Yes vs. no | 2.26 (1.59-3.22) | <0.001 | 1.68 (1.11-2.55) | 0.014 | 1.86 (1.25-2.76) | 0.002 | 1.58 (1.03-2.42) | 0.038 |
| Bone metastasis | | Yes vs. no | 1.80 (1.37-2.37) | <0.001 | 1.54 (1.14-2.08) | 0.005 | 1.86 (1.38-2.52) | <0.001 | 1.62 (1.17-2.25) | 0.004 |
| Hypertension | | Yes vs. no | 0.98 (0.74-1.29) | 0.869 |  |  | 1.07 (0.78-1.47) | 0.661 |  |  |
| COPD | | Yes vs. no | 0.94 (0.70-1.26) | 0.677 |  |  | 1.13 (0.81-1.56) | 0.473 |  |  |
| Diabetes | | Yes vs. no | 1.05 (0.75-1.49) | 0.770 |  |  | 1.21 (0.83-1.77) | 0.323 |  |  |
| Cardiovascular disease | | Yes vs. no | 1.48 (1.03-2.13) | 0.035 | 1.71 (1.18-2.48) | 0.004 | 1.94 (1.32-2.85) | 0.001 | 2.21 (1.47-3.32) | <0.001 |
| CKD | | Yes vs. no | 1.15 (0.57-2.33) | 0.690 |  |  | 0.95 (0.42-2.15) | 0.910 |  |  |
| NLR | | Ratio | 1.03 (1.00-1.07) | 0.057 | 1.02 (0.97-1.07) | 0.410 | 1.03 (1.00-1.07) | 0.070 | 1.03 (0.99-1.07) | 0.119 |
| PLR | | Ratio | 1.00 (1.00-1.00) | 0.016 | 1.00 (1.00-1.00) | 0.459 | 1.00 (1.00-1.00) | 0.103 |  |  |
| LDH | | UI/L | 1.00 (1.00-1.00) | 0.006 | 1.00 (1.00-1.00) | 0.565 | 1.00 (1.00-1.00) | 0.018 | 1.00 (1.00-1.00) | 0.929 |
| SCS (time-varying) | | SCS vs. no steroids | 2.01 (1.42-2.84) | <0.001 | 1.99 (1.40-2.84) | <0.001 | 1.89 (1.36-2.62) | <0.001 | 1.77 (1.25-2.51) | 0.001 |
| ICS (time-varying) | | ICS vs. no steroids | 1.17 (0.58-2.40) | 0.657 | 1.35 (0.66-2.80) | 0.412 | 1.46 (0.74-2.86) | 0.272 | 1.48 (0.74-2.97) | 0.269 |
| Abbreviations: PFS, progression-free survival; OS, overall survival; HR, hazard ratio; CI, confidence interval; BMI, body mass index; ECOG PS, Eastern Cooperative Oncology Group Performance Status; PD-L1, programmed cell death ligand 1; ICI, immune checkpoint inhibitor; PD-1, programmed cell death 1; COPD, chronic obstructive pulmonary disease; CKD, chronic kidney disease; NLR, neutrophil-to-lymphocyte ratio; PLR, platelet-to-lymphocyte ratio; LDH, lactate dehydrogenase; SCS, systemic corticosteroids; ICS, inhaled corticosteroids.  ^*^PD-L1 positive was defined as PD-L1 TPS ≥1%. | | | | | | | | | | |

**Legends of figures**

**Figure 1 Participants enrollment flow diagram.**

Abbreviations: ICI, immune checkpoint inhibitor; PD-1, programmed cell death 1; PD-L1, programmed cell death ligand 1; SCS, systemic corticosteroids; ICS, inhaled corticosteroids.

**Figure 2 Clinical outcomes of patients treated with concurrent SCS, ICS, and no steroids treatment.**

(A) Kaplan-Meier curves for progression-free survival. (B) Kaplan-Meier curves for overall survival. (C) Objective response rate. (D) Disease control rate. Abbreviations: SCS, systemic corticosteroids; ICS, inhaled corticosteroids; HR, hazard ratio; CI, confidence interval; CR, complete response; PR, partial response; SD, stable disease; PD, progressive disease.

**Figure 3 Forest plots of subgroup analyses for OS according to CS prescription based on time-dependent Cox regression.**

Subgroup analyses were based on time-dependent Cox models adjusted for age and PD-L1 status. Abbreviations: HR, hazard ratio; CI, confidence interval; ICI, immune checkpoint inhibitor; PD-1, programmed cell death 1; PD-L1, programmed cell death ligand 1; TPS, tumor proportion score; COPD, chronic obstructive pulmonary disease; SCS, systemic corticosteroids; ICS, inhaled corticosteroids.
